# Rapid and sustained degradation of the essential centrosome protein CEP192 in live mice using the AID2 system

**DOI:** 10.1126/sciadv.adq2339

**Published:** 2025-02-28

**Authors:** Valentina C. Sladky, Margaret A. Strong, Daniel Tapias-Gomez, Cayla E. Jewett, Chelsea G. Drown, Phillip M. Scott, Andrew J. Holland

**Affiliations:** Department of Molecular Biology and Genetics, Johns Hopkins University School of Medicine, Baltimore, MD 21205, USA.

## Abstract

Studying essential genes required for dynamic processes in live mice is challenging as genetic perturbations are irreversible and limited by slow protein depletion kinetics. The auxin-inducible degron (AID) system is a powerful tool for analyzing inducible protein loss in vitro, but it is toxic to mice. Here, we use an optimized second-generation AID system to achieve the conditional and reversible loss of the essential centrosomal protein CEP192 in live mice. We show that the auxin derivative 5-phenyl-indole-3-acetic acid is well tolerated over 2 weeks and drives near-complete CEP192 degradation in less than 1 hour in vivo. CEP192 loss did not affect centriole duplication but decreased γ-tubulin recruitment to centrosomes impairing mitotic spindle assembly. Sustained CEP192 loss in vivo led to cell division failure and cell death in proliferative tissues. Thus, the second-generation AID system is well suited for rapid and/or sustained protein depletion in live mice to study essential functions in vivo.

## Teaser

AID2-mediated degradation of CEP192 in live mice reveals a critical role in mitosis but not centriole duplication or ciliogenesis.

## INTRODUCTION

Loss-of-function mutant mice are critical parts of the genetic toolbox for basic research. However, DNA- and RNA-based knockout and knockdown approaches have limitations, including irreversibility and slow depletion dynamics. These shortcomings can be overcome by induced degradation with chemical methods such as PROTACs (Proteolysis Targeting Chimeras) (*1*) or chemical genetic approaches like the dTAG system or auxin-inducible degron (AID) system (*2*–*4*). PROTACs are bivalent binders that bridge the protein of interest and an E3 ligase to promote proximity-induced ubiquitination (*5*, *6*). PROTACs require the engineering of chemical ligands that specifically bind the target protein, limiting their applications to ligandable targets. The dTAG system requires tagging the gene of interest with an FKBP12^F36V^ tag so that a heterobifunctional degrader can recruit the FKBP12^F36V^-tagged target to cereblon (CRBN) for ubiquitination and degradation (*7*). dTag has proven valuable for short-term applications in mice, achieving near-complete protein depletion in xenograft tumors, adult tissues, and embryos (*8*–*10*). However, long-term applications in mice are limited by the toxicity of the vehicle and the small molecule required to induce protein degradation (*11*). In addition, PROTACs and the dTAG system are based on heterobifunctional molecules that can display a Hook effect due to the saturated binding of the ligase and degradation target at high concentrations (*12*).

The AID system uses the plant hormone auxin, which acts as a molecular glue to stabilize the binding of an AID to the plant-derived E3 ligase adaptor Tir1 (transport inhibitor response 1). In the presence of the auxin hormone indole-3-acetic acid (IAA), the TIR1-SCF E3 ligase complex targets AID-tagged proteins for degradation (*13*). The AID system has been applied successfully in mammalian cells to drive inducible protein degradation, reducing the half-life of targeted proteins to <30 min (*13*, *14*). However, the original AID system suffers from leakiness and requires high IAA concentrations (100 to 500 μM). These limitations were overcome with the development of a second-generation AID system (AID2), in which a point mutation in *Tir1* (F74A or F74G) promotes an interaction with the bulky IAA derivatives 5-adamantyl-indole-3-acetic acid (5-Ad-IAA) or 5-phenyl-indole-3-acetic acid (5-Ph-IAA) at up to 1000-fold lower concentrations (*15*). The AID2 system has been shown to work for in vivo applications in *Drosophila melanogaster* (*16*) and *Caenorhabditis elegans* (*17*).

Recent efforts have focused on applying the AID system for in vivo use in mice (*18*–*20*). The first-generation AID system has been used to target CDC7 (*20*) and the condensin subunits NCAPH and NCAPH2 (*19*) for degradation in mice expressing *Oryza sativa Tir1* (*OsTir1*) from the *Rosa26* locus. Both studies report protein depletion within 2 to 6 hours in the cell types tested (*19*, *20*). However, in vivo IAA treatment caused notable toxicity in mice; therefore, most experiments were performed ex vivo (*19*, *20*). A recent proof-of-principle study tested the second-generation AID system in mice expressing a randomly integrated *OsTir1*-F74G transgene. Injections of the auxin derivative 5-Ph-IAA achieved robust protein degradation within 6 hours in various tissues. However, these results were limited to analyzing the degradation of a randomly integrated GFP (green fluorescent protein) reporter carrying an AID tag, and long-term 5-Ph-IAA treatments or pathological assessments were not performed (*18*).

The AID system has been widely used in cultured cell lines to study essential genes in cellular processes such as mitosis and centrosome biology (*14*, *21*–*24*); however, it has not yet been applied to analyzing essential genes in mice. CEP192 is a centrosomal protein characterized as a common essential gene by the Cancer Dependency Map (DepMap). The centrosome is a membraneless organelle consisting of a pair of centrioles surrounded by pericentriolar material (PCM) and functions as a microtubule organizing center in some interphase cells. In quiescent cells, modified centrioles function as basal bodies that template cilia critical for signaling, fluid transport, and locomotion (*25*). Centrioles are duplicated once during S phase to ensure their numbers are maintained from one generation to the next (*26*). This process is controlled by the kinase Polo-like kinase 4 (PLK4), which is recruited to the existing parent centrioles by CEP152 and CEP192 (*26*–*29*). Whether CEP192 is required for centriole duplication in mammalian cells is controversial with some studies reporting centriole duplication defects in CEP192 depleted cells and others not (*28*–*31*). The parent centrioles mature during mitotic traverse and acquire appendages needed for signaling and ciliogenesis (*25*, *32*–*34*). During mitosis, the centrosome also undergoes maturation and expands the PCM to increase microtubule nucleation and establish the bipolar mitotic spindle apparatus (*26*). CEP192 serves as a scaffold for PCM recruitment, including the γ-tubulin ring complex that is needed for microtubule nucleation (*31*, *35*–*37*).

Almost all the analysis of mammalian centrosomes and CEP192 has been performed in cultured cells. However, recent works have shown that centrosome composition and function can vary across mammalian tissues and cell types (*38*–*41*). Moreover, analyzing CEP192 function in mouse models in vivo is challenged by the fast dynamics of centriole assembly and cell division. In addition, the interconnected roles of centrioles in ciliogenesis, centrosome assembly, and cell division have made it challenging to discern the direct role of CEP192 in these processes. Here, we use the second-generation AID system in live mice to investigate the in vivo functions of the essential centrosome protein CEP192.

## RESULTS

### Creation of CEP192-mAID-mNeonGreen and conditional OsTir1-F74G mouse lines

**Fig. 1.**
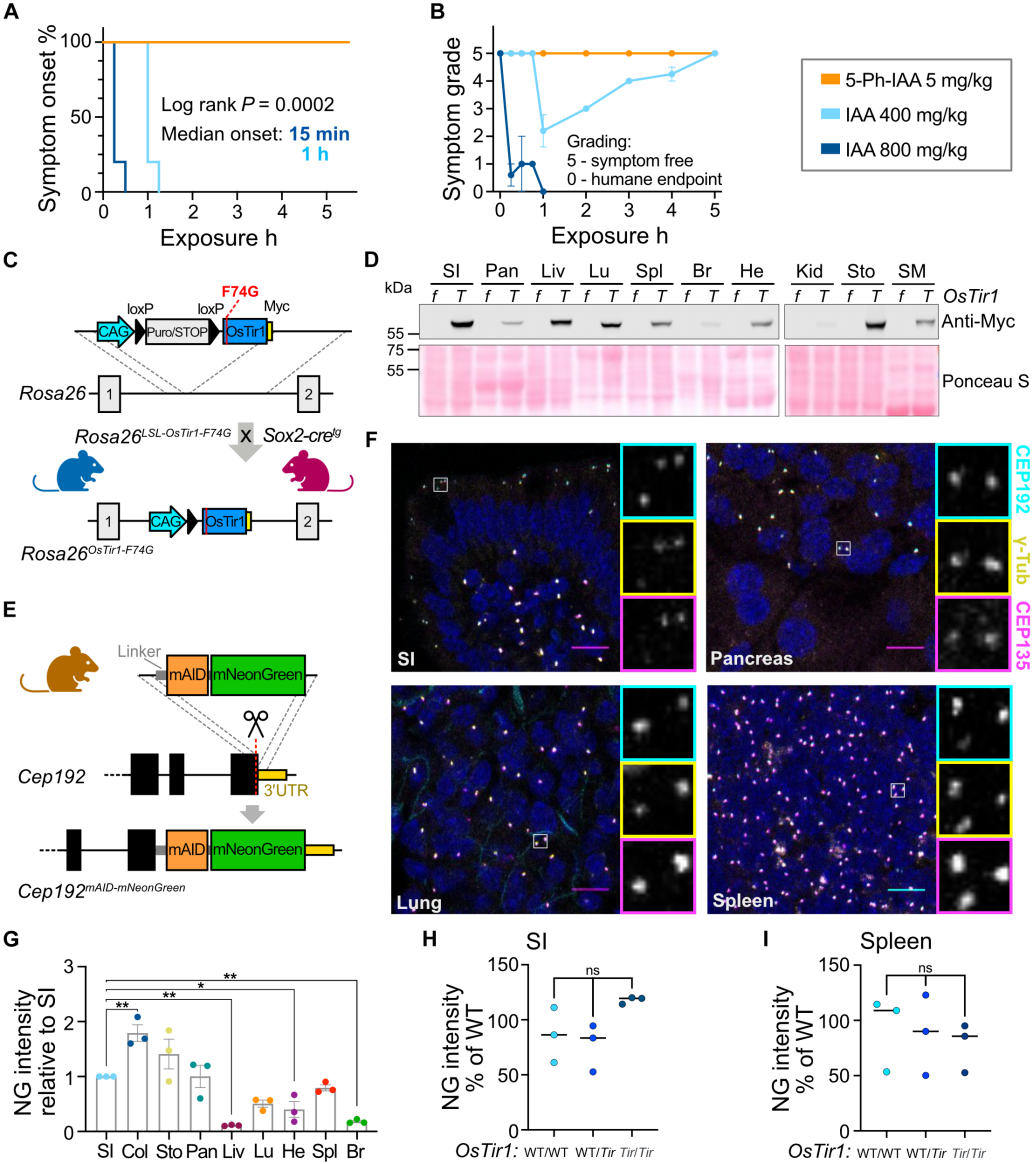
Characterization of *OsTir1*-*F74G* and *Cep192*^*mAID*-*mNG*^ mice reveals robust expression across tissues. (**A**) Kaplan-Meier plot showing symptom onset after intraperitoneal injection of *OsTir1^Tir/Tir^* mice with 5-Ph-IAA (5 mg/kg; *N* = 3), IAA (400 mg/kg; *N* = 5), or IAA (800 mg/kg; *N* = 5). h, hours. (**B**) Symptoms were graded by severity: 5, symptom-free; 0, near-complete paralysis, humane endpoint. 5-Ph-IAA (5 mg/kg; *N* = 3), IAA (400 mg/kg; *N* = 5), or IAA (800 mg/kg; *N* = 5). (**C**) Schematic showing the targeting strategy to integrate the lox-stop-lox (LSL)-*OsTir1-Myc* construct at the *Rosa26* locus. (LSL)-*OsTir1-Myc* animals were crossed to Sox2-Cre expressing mice to achieve whole-body expression of OSTIR1-F74G-Myc. (**D**) Immunoblot probed with an antibody detecting Myc-tagged OSTIR1 in the noted organs isolated from an *OsTir1^f/f^* (*f*) and an *OsTir1^Tir/Tir^* (*T*) mouse. SI, small intestine; Pan, pancreas; Liv, liver; Lu, lung; Spl, spleen; Br, brain; He, heart; Kid, kidney; Sto, stomach; SM, skeletal muscle. Ponceau S staining is shown as a loading control. (**E**) Strategy to target exon 48 of *Cep192* to endogenously tag the C terminus of *Cep192* with mAID and moxNeonGreen (*Cep192^AID^*). (**F**) Representative immunofluorescence images of the indicated organs showing CEP192^AID^ signal (NeonGreen; cyan), and immunostained γ-tubulin (yellow) and CEP135 (magenta); Scale bars, 10 μm. (**G**) Quantification of the CEP192^AID^ NeonGreen (NG) signal intensity in the noted tissues was measured by fluorescence microscopy and is shown relative to the SI. Col, colon; Sto, stomach; Pan, pancreas; Liv, liver; Lu, lung; He, heart; Spl, spleen; Br, brain. *N* = 3 mice; *n* = 40 to 100 cells per mouse. (**H** and **I**) Graphs showing the NeonGreen (NG) signal quantified in the SI (H) and the spleen (I) of untreated *Cep192^AID^* mice that were either WT/WT, WT/*Tir*, or *Tir/Tir* for *OsTir1*. *N* = 3 mice per genotype; *n* = 50 to 100 cells per mouse. Data are shown as means ± SEM. Statistical significance was determined using one-way ANOVA with Sidak’s multiple comparisons test [(G) to (I)]. ns *P* ≥ 0.05; *P < 0.05; **P < 0.01. (C) and (E) were partially created using Biorender.com.

To evaluate the utility of the first-generation AID system in mice, we first examined the effects of administering IAA at doses used previously to achieve protein degradation in mice. Intraperitoneal injection of a single dose of IAA at 400 or 800 mg/kg in phosphate-buffered saline (PBS) induced spasms and paralysis within 1 hour or 15 min, respectively (Fig. 1A and fig. S1A). Although some mice dosed with 400 mg/kg recovered, all animals receiving 800 mg/kg reached the humane endpoint with near-complete paralysis after 1 hour (Fig. 1B and fig. S1A). We conclude that IAA is toxic in mice at the doses required to induce protein degradation (fig. S1B), and thus, the first-generation AID system is unsuitable for the in vivo manipulation of protein levels.

We next analyzed the effect of the IAA derivative 5-Ph-IAA that is used in the AID2 system at similar doses to those used previously in mice (*18*). Mice injected with 5-Ph-IAA (5 mg/kg) in PBS were symptom-free over the observation period of 5 hours (Fig. 1A and fig. S1A). This motivated us to develop mouse models to test the second-generation AID system in vivo. We created a conditional allele of *OsTir1-F74G* by targeting a loxP-Stop-loxP cassette followed by *OsTir1-F74G-Myc* to the *Rosa26* locus. The resulting *Rosa26-OsTir1-F74G^flox^* mice were crossed to *Sox2-Cre* animals that express Cre in the germ line to generate *Rosa26-OsTir1-F74G^Tir^* animals, hereafter referred to as *OsTir1^Tir^* (Fig. 1C). *OsTir1^Tir^* animals had a similar body weight to *OsTir1^flox^* mice (fig. S1C). Homozygous *OsTir1^Tir/Tir^* mice were fertile, and heterozygous breedings produced offspring at Mendelian ratios (fig. S1, D and E). The OSTIR1 protein was expressed across various tissues, with the lowest expression detected in the brain and kidney (Fig. 1D and fig. S1, F and G).

To test the effectiveness of *OsTir1*-F74G mediated protein degradation, we endogenously tagged the C-terminal exon of *Cep192* with a flexible linker, a miniaturized AID tag [mAID; (*42*)], and moxNeonGreen to track protein abundance (Fig. 1E). Heterozygous and homozygous *Cep192^mAID-moxNeonGreen^* mice (hereafter *Cep192^AID^*) are fertile and produce offspring at the expected Mendelian ratios (fig. S1, D and H). Body weights of the various combinations of *Cep192^AID^*; *OsTir1^Tir^* genotypes were similar to *OsTir1^flox^* mice (fig. S1C), indicating that tagging did not interfere with the essential roles of CEP192. Immunofluorescence analysis showed that endogenously tagged CEP192 (hereafter CEP192^AID^) was expressed across various tissues and colocalized with the centrosomal proteins CEP135 and γ-tubulin (Fig. 1, F and G, and fig. S1, I to K). Although CEP192^AID^ abundance at the centrosome was similar across cell types in most tissues, in the liver, hepatocytes displayed minimal CEP192^AID^ expression and CEP192^AID^ was only detected in nonparenchymal liver cells (fig. S1, I and J). Leaky degradation was not observed in the absence of 5-Ph-IAA in the small intestine (SI) and spleen of *Cep192^AID/AID^* animals heterozygous or homozygous for *OsTir1^Tir^* (Fig. 1, H and I). Together, we conclude that 5-Ph-IAA can overcome the toxicity-related limitations of IAA, making the AID2 system suitable for use in vivo. Moreover, we find that the homozygous *OsTir1*-*F74G* and *Cep192^AID^* alleles are well tolerated and widely expressed in mice.

### The AID2 system is suitable for long-term repeat dose experiments in mice

**Fig. 2.**
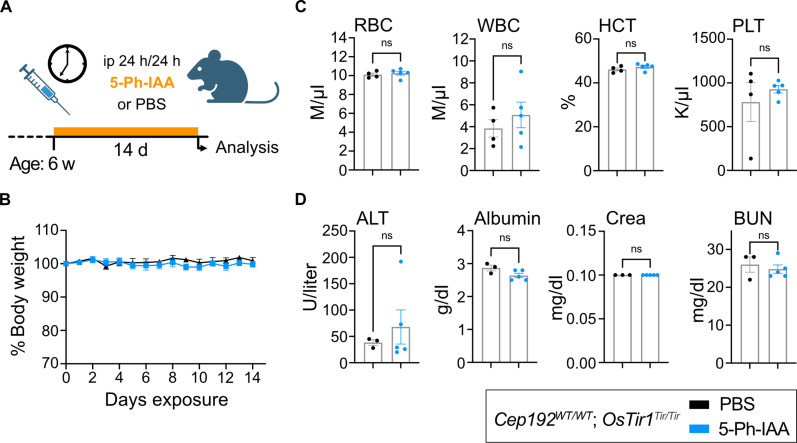
Repeated dosing with 5-Ph-IAA is well tolerated over time. (**A**) Schematic illustration of the treatment plan. Six-week-old *OsTir1^Tir/Tir^* mice were injected intraperitoneally (ip) with 5-Ph-IAA or PBS every 24 hours for 14 days. d, days; w, weeks; h, hours. (**B**) Graph showing body weight changes of mice treated as described in (A) and monitored over the treatment period. *N* = 6 mice per treatment. (**C** and **D**) Blood cell counts and serum parameters indicating toxicity were measured after 14 days of treatment. Full panels are shown in fig. S2. *N* = 6 mice per treatment. (C) Blood cell counts: RBC, red blood cells; WBC, white blood cells; HCT, hematocrit; PLT, platelet count. (D) Serum parameters: ALT, alanine aminotransferase; Crea, creatinine; BUN, blood urea nitrogen. Data are displayed as means ± SEM. Statistical significance was assessed by a two-tailed, unpaired Student’s *t* test; ns *P* ≥ 0.05. (A) was partially created using Biorender.com.

Because a single dose of 5-Ph-IAA was well tolerated, we next tested the impact of chronic 5-Ph-IAA administration in mice. We injected *Cep192^WT/WT^*; *OsTir1^Tir/Tir^* mice (*n* = 6, three females and three males) every 24 hours for 14 days with 5-Ph-IAA (5 mg/kg) or vehicle control (PBS; Fig. 2A). Daily monitoring of animal behavior and body weight showed no abnormalities (Fig. 2B). After the last injection, a full necropsy was performed and over 24 tissues were evaluated by a board-certified veterinary pathologist. 5-Ph-IAA treatment did not affect any of the tissues examined. Complete blood cell counts and serum analysis were also indistinguishable between the PBS- or 5-Ph-IAA–treated groups (Fig. 2, C and D, and fig. S2, A and B). This shows that repeated injections with an effective concentration of 5-Ph-IAA are well tolerated over at least 14 days.

### AID2 induced rapid and reversible CEP192^AID^ degradation in vivo

**Fig. 3.**
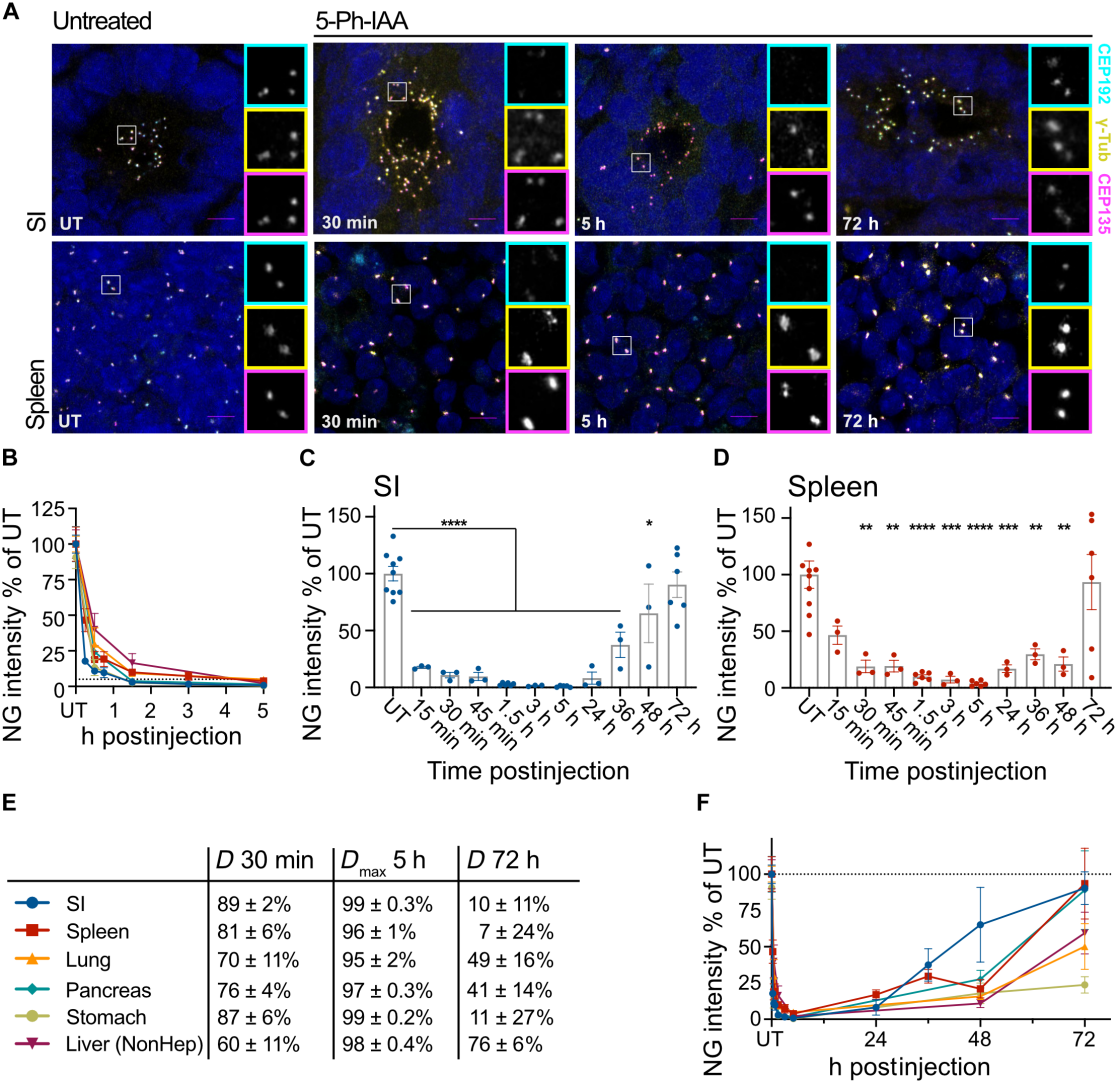
The AID2 system induces rapid and reversible CEP192^AID^ degradation in mice. (**A**) Representative confocal images of the SI and the spleen of *CEP192^AID^* mice expressing OSTIR1 that were left untreated (UT) or analyzed at various times after intraperitoneal injection of 5-Ph-IAA. CEP192^AID^ (cyan), γ-tubulin (yellow), and CEP135 (magenta); scale bars, 5 μm. (**B**) Graph showing the CEP192^AID^ intensity [NeonGreen (NG)] measured by fluorescence microscopy relative to the untreated (UT) control in the SI (blue), spleen (red), lung (orange), pancreas (teal), stomach (green), and nonparenchymal liver cells (NonHep, purple). Mice were analyzed at 15 min (SI and spleen only), 30 min and 1.5, 3 (SI and spleen only), and 5 hours after intraperitoneal injection of 5-Ph-IAA (5 mg/kg). (**C** and **D**) Bar graphs showing the individual data points of the CEP192^AID^ [NeonGreen (NG)] signal intensity quantifications in the SI (C) and the spleen (D) relative to the untreated controls (UT) at the indicated time points. (**E**) Table shows the degree of degradation at 30 min and 5 and 72 hours post–5-Ph-IAA injection for the indicated organs. Maximum degradation (*D*_max_) was reached by 5 hours in all organs tested. (**F**) The CEP192^AID^ signal (NG) was quantified at the indicated time points up to 72 hours after 5-Ph-IAA injection. Data points for UT and time points up to 5 hours are the same as in (B). The dashed line indicates 100%. Individual data points for the lung, pancreas, stomach, and liver are shown in fig. S3. *N* = 3 to 9 mice per time point with *n* = 40 to 100 cells analyzed per mouse. Data are shown as means ± SEM. Statistical significance was determined using a one-way ANOVA with Sidak’s multiple comparisons test comparing each time point to the untreated control. *P < 0.05; **P < 0.01; ***P < 0.001; ****P < 0.0001. Only significant results are indicated. h, hours.

We next examined the in vivo degradation dynamics of CEP192^AID^ in different tissues. *Cep192^AID/AID^*; *OsTir1^Tir/Tir^* mice were injected intraperitoneally with 5-Ph-IAA (5 mg/kg) in PBS, and CEP192^AID^ signal intensity (NeonGreen) was analyzed by fluorescence microscopy at various time points. Thirty minutes after 5-Ph-IAA administration, CEP192^AID^ was significantly reduced in all tissues tested with the highest degradation rate in the SI (Fig. 3, A to E, and fig. S3, A to E). At 1.5 hours after injection, >90% CEP192^AID^ was degraded in the SI, spleen, lung, pancreas, and stomach (Fig. 3, A to E, and fig. S3, A to E). The degradation maximum (*D*_max_) reached over 95% in all tissues analyzed at 5 hours after 5-Ph-IAA injection, with the SI, pancreas, and stomach showing ~99% CEP192^AID^ degradation (Fig. 3, A to E, and fig. S3, A to E). The CEP192^AID^ signal remained low for 24 hours after 5-Ph-IAA administration and showed almost complete recovery by 72 hours postinjection in the SI, spleen, and pancreas (Fig. 3, A and C to F, and fig. S3, A to E). CEP192^AID^ recovery dynamics were slower in the other tissues reaching ~40% of control CEP192^AID^ levels in the lung and nonparenchymal liver cells and ~30% in the stomach at 72 hours post–5-Ph-IAA injection (Fig. 3, E and F, and fig. S3, A to E). We also tested the effect of a lower (1 mg/kg) dose of 5-Ph-IAA (fig. S3, F and G). Treatment with 1 mg/kg degraded ~80% of CEP192^AID^ in the SI but only reduced CEP192^AID^ by 53% in the spleen (fig. S3, F and G). Together, these data show that the AID2 system allows reversible, near-complete degradation of CEP192^AID^ in less than 5 hours across different tissues, and degradation can be tuned with the dose of 5-Ph-IAA used.

### CEP192 is required for bipolar spindle assembly in mitosis but not centriole duplication

**Fig. 4.**
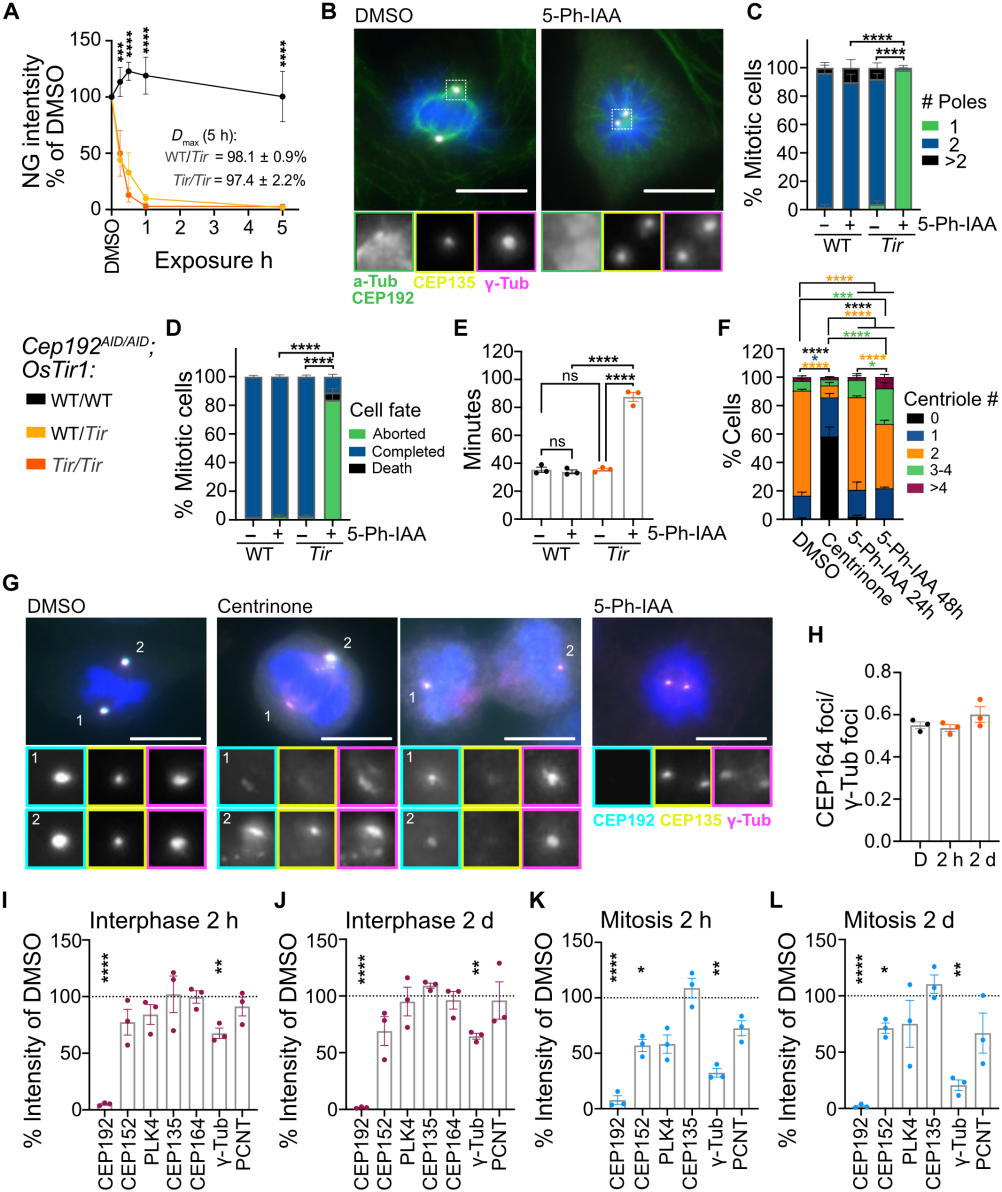
CEP192 is required to establish a bipolar mitotic spindle but not for centriole duplication. (**A**) Quantification of NeonGreen (NG) intensity relative to DMSO in MEFs treated with 5-Ph-IAA for 15 and 30 min and 1 and 5 hours. *N* = 3 to 5 MEF lines per genotype; *n* = 50 to 100 cells per MEF line. (**B** and **C**) *Cep192^AID/AID^* MEFs were treated with 5-Ph-IAA (+) or DMSO (−) for 5 hours. (B) Representative images of OSTIR1 expressing MEFs. Insets show centrosomes in mitotic cells. (C) Graph showing quantification of the number of mitotic spindle poles; *n* = 10 to 30 mitotic cells in *N* = 4 MEF lines per genotype. (**D** and **E**) Primary *Cep192^AID/AID^* MEFs with or without OSTIR1 expression were treated with 5-Ph-IAA (+) or DMSO (−) and live imaged for 48 hours. (D) Graph showing cell fate after mitosis. (E) Quantification of the time spent in mitosis. *n* = 50 to 100 mitotic cells per *N* = 4 MEF lines per genotype. (**F** and **G**) SV40-immortalized *Cep192*^*AID*/*AID*^; *OsTir1^Tir/Tir^* MEF lines were treated with centrinone (48 hours) and 5-Ph-IAA (24 and 48 hours) and centriole numbers counted using CEP135 foci. (F) Stacked bar graphs show centriole number distribution. (G) Representative micrographs of mitotic cells. Insets show centrosomes of both spindle poles. (**H**) Bar graph shows the ratio of CEP164 foci to γ-tubulin foci per cell in SV40-immortalized *Cep192*^*AID*/*AID*^; *OsTir1^Tir/Tir^* MEFs treated with DMSO (D) or 5-Ph-IAA for 2 hours and 2 days. (**I** to **L**) Abundance of centrosome proteins in SV40-immortalized *Cep192*^*AID*/*AID*^; *OsTir1^Tir/Tir^* MEFs treated with DMSO or 5-Ph-IAA for 2 hours and 2 days. Signal intensities were measured in interphase [(I) and (J)] and mitotic cells [(K) and (L)]. *N* = 3 MEF lines with *n* = 50 to 100 interphase cells and *n* = 5 to 20 mitotic cells per MEF line and condition. Scale bars, 10 μm. All data are displayed as means ± SEM. Statistical significance was determined using one-way [(E) and (I) to (L)] or two-way [(A), (C), (D), and (F)] ANOVA with Sidak’s multiple comparisons test. Only significant results are indicated. ns *P* ≥ 0.05; *P < 0.05; **P < 0.01; ***P < 0.001; ****P < 0.0001. h, hours; d, days.

To explore the function CEP192 in centriole duplication and spindle assembly, we generated primary *Cep192^AID/AID^* mouse embryonic fibroblast (MEF) lines from embryos WT/WT, *Tir*/WT, or *Tir*/*Tir* for *OsTir1*. We first analyzed the dynamics of CEP192^AID^ degradation by measuring the abundance of CEP192^AID^ at the centrosome using immunofluorescence. Treatment with 1 μM 5-Ph-IAA led to near-complete degradation of centrosomal CEP192^AID^ within 1 hour in both heterozygous and homozygous *OsTir1* lines (Fig. 4A). The *D*_max_ was 98% for WT/*Tir* and 97% for *Tir*/*Tir* genotypes (Fig. 4A). We further analyzed CEP192^AID^ degradation dynamics using live imaging and confirmed efficient degradation of CEP192^AID^ within 1 hour in both *OsTir1* homozygous and heterozygous cells (fig. S4A). Costaining with an antibody raised against murine CEP192 confirmed complete protein degradation at 5 hours after 5-Ph-IAA addition (fig. S4B). Thus, CEP192^AID^ can be effectively degraded using the AID2 system in MEFs.

5-Ph-IAA–treated *Cep192^AID/AID^* MEF cells expressing OSTIR1 almost exclusively formed monopolar spindles during mitosis (Fig. 4, B and C), and live imaging showed that over 80% of MEFs lacking CEP192^AID^ delayed in mitosis and failed to divide before readhering to the plate (Fig. 4, D and E, and fig. S4C). For further experiments, we used SV40-immortalized *Cep192^AID/AID^*; *OsTir1^Tir/Tir^* MEF lines that lack a functional TP53 response. Similar to primary MEFs, 5-Ph-IAA treatment of SV40-immortalized *Cep192^AID/AID^*; *OsTir1^Tir/Tir^* MEFs for 24 hours delayed mitosis and resulted in ~60% of cells failing to divide (fig. S4, D and E). Washout of 5-Ph-IAA for another 24 hours led to an ~25% recovery of CEP192^AID^ that rescued mitotic timing and successful cell division (fig. S4, D to F).

To assess whether murine CEP192 is required for centriole duplication, we treated immortalized *Cep192^AID/AID^*; *OsTir1^Tir/Tir^* MEFs for 24 and 48 hours with 5-Ph-IAA to deplete CEP192^AID^ or with the PLK4 inhibitor centrinone to block centriole duplication. The number of centrioles was measured by counting the number of CEP135 foci by immunofluorescence (Fig. 4, F and G). Although 2 days of centrinone treatment caused a depletion of centrioles, CEP192^AID^ degradation did not affect centriole numbers at 24 hours and resulted in an increase in centriole number by 2 days (Fig. 4F and fig. S4G). This is likely because cells lacking CEP192^AID^ undergo a high rate of mitotic failure, leading to the generation of polyploid cells with extra centrioles (Fig. 4F and fig. S4, G and H). Centriole depletion by centrinone treatment caused a small and nonsignificant increase in mitotic duration, and most cells completed a normal bipolar cell division (fig. S4, D and E). CEP192^AID^ localized to the acentriolar mitotic spindle poles of centrinone-treated MEFs lacking centrioles (Fig. 4G).

To assess the impact of acute and chronic CEP192 loss on centrosome function, we treated immortalized *Cep192^AID/AID^*; *OsTir1^Tir/Tir^* MEFs with 5-Ph-IAA for 2 hours or 2 days and measured the abundance of different centriolar and centrosomal proteins. Centriole integrity and maturation were assessed by monitoring the centriole protein CEP135 and the distal appendage protein CEP164 (*25*, *26*). CEP164 is present at mature parent centrioles and is expected to be recruited to 50% of interphase centrosomes (*25*). Competence for centriole duplication was assessed by measuring PLK4, CEP152, and CEP192 levels. PLK4 is essential for centriole duplication and is recruited to the centrioles by CEP152 and CEP192 (*28*, *29*). We also measured the abundance of γ-tubulin and pericentrin (PCNT) to evaluate PCM assembly. γ-Tubulin is required for microtubule nucleation at the centrosome to form a bipolar spindle (*35*), whereas PCNT acts upstream of CEP192 in recruiting PCM components (*36*).

None of the centriole proteins examined were affected by acute or long-term CEP192^AID^ loss in interphase cells (Fig. 4, H to J, and fig. S4I). The presence of CEP164 on ~50% of centrosomes suggests that centriole maturation was not affected by the absence of CEP192^AID^ (Fig. 4H). CEP192^AID^ degradation reduced the abundance of CEP152 and PLK4 by ~40% in mitosis, but these proteins were unaffected in interphase cells, explaining why centriole duplication was not altered by CEP192^AID^ degradation (Fig. 4, I to L). PCNT abundance was not significantly affected by CEP192^AID^ loss in interphase or mitosis. However, short-term and long-term degradation of CEP192^AID^ significantly reduced centrosomal γ-tubulin by ~25% in interphase (Fig. 4, I and J) and ~75% in mitosis (Fig. 4, K and L, and fig. S4I). This is in accord with the impaired bipolar spindle formation observed in cells lacking CEP192 (Fig. 4, B and C). Together, our data suggest that, in mice, CEP192 has a critical role in centrosome maturation and not centriole duplication.

### CEP192 is not required for the formation or maintenance of primary or motile cilia

**Fig. 5.**
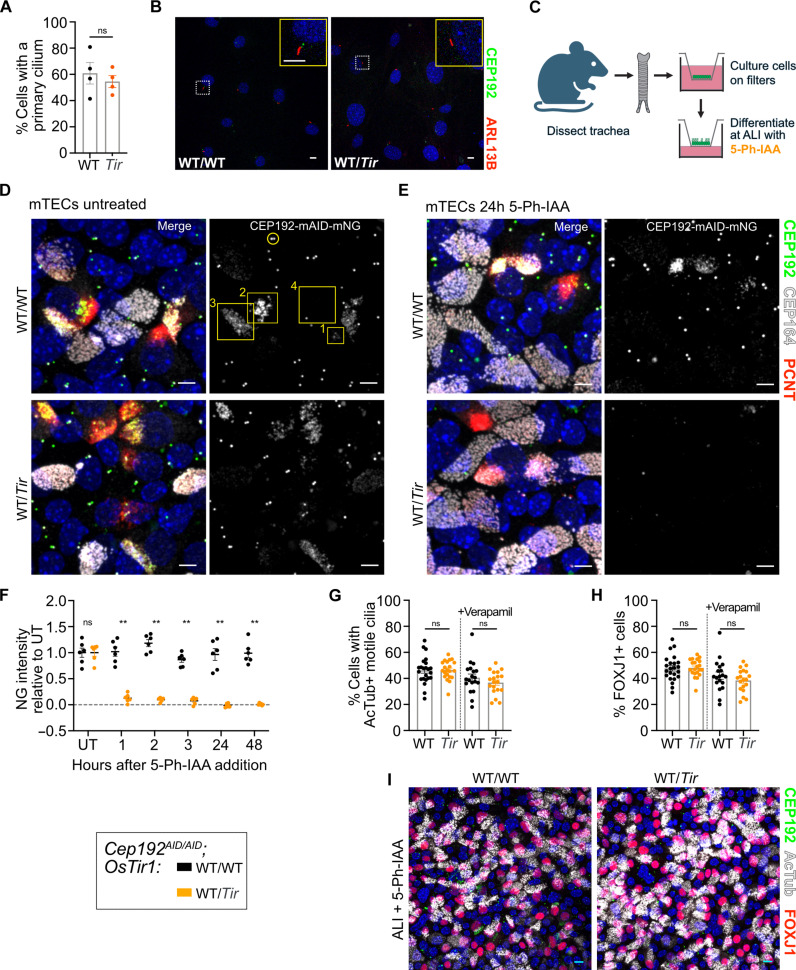
CEP192 is not required for the formation or maintenance of primary or motile cilia. (**A** and **B**) *Cep192^AID/AID^* MEF lines WT/WT or Tir/Tir for *OsTir1* were serum starved in the presence of 5-Ph-IAA for 24 hours. (A) Quantification of the number of cells with a primary cilium; *N* = 2 MEF lines per genotype. (B) Representative confocal image of MEFs expressing CEP192^AID^ (green) and immunostained for the primary cilia marker Arl13B (red). Insets show a primary cilium. (**C**) Schematic showing isolation and differentiation of mTECs. (**D**) Representative confocal images of mTECs at ALI day 5. Yellow circle marks the CEP192^AID^ signal at the centrosome in nondifferentiating cells. Yellow boxes mark the CEP192^AID^ signal in differentiating cells with numbers denoting early (1), mid (2), and late (3), and complete (4) differentiation. (**E**) Representative confocal images of mTECs at ALI day 5 treated with 5-Ph-IAA for 24 hours. (**F**) Time course showing CEP192^AID^ intensity at newly amplified centrioles following 5-Ph-IAA treatment in differentiating cells. The signal intensity was normalized to the untreated (UT) condition. *N* = 2 mice per genotype indicated by different symbols; *n* = 6 fields of view. (**G** to **I**) mTECs expressing CEP192^AID^ were treated with 5-Ph-IAA for ALI days 0 to 7. mTEC cultures were immunostained for the differentiation marker FOXJ1 and for acetylated tubulin to label motile cilia (AcTub). (G) Percentage of cells with motile cilia +/− Verapamil. (H) Percentage of cells in with FOXJ1+ nuclei +/− Verapamil. [(G) and (H)] *N* = 3 mice per genotype; n = 6 fields of view per genotype and condition. (I) Representative confocal image of mTEC cultures on ALI day 7. Scale bars, 5 μm. All data are displayed as means ± SEM. Statistical significance was determined using two-way ANOVA with Sidak’s multiple comparisons test (F) or a two-tailed, unpaired Student’s *t* test [(A), (G), and (H)]. ns *P* ≥ 0.05; **P < 0.01.

The requirement of CEP192 for centrosome function has prevented a clear analysis of its role in ciliogenesis. We therefore exploited the rapid depletion of CEP192^AID^ in primary MEFs to evaluate CEP192’s role in ciliogenesis. Primary *Cep192^AID/AID^* MEF lines with or without *OsTir1* were serum starved for 24 hours in the presence of 5-Ph-IAA. The number of cells with a primary cilium was unaffected by CEP192^AID^ degradation before ciliogenesis (Fig. 5, A and B, and fig. S5A). Similarly, degradation of CEP192^AID^ for 24 or 48 hours after the primary cilium was established did not alter the number of ciliated cells (fig. S5, B and C). We conclude that CEP192 is dispensable for the formation and maintenance of the primary cilium.

To further evaluate the role of CEP192 in ciliogenesis, we used ex vivo cultures of mouse tracheal epithelial cells (mTECs), which differentiate into multiciliated cells (MCCs) that amplify centrioles to build hundreds of motile cilia at their apical cell surface. To generate mTECs, tracheas were dissected from *Cep192^AID/AID^* mice with or without *OsTir1* and cultured on transwell filters to form a polarized epithelial monolayer. Cells were then differentiated at an air-liquid interface (ALI), and 5-Ph-IAA was added to media in the basal chamber at various time points (Fig. 5C). The resulting culture contains several cell types, including MCCs. CEP192^AID^ localized to centrosomes of non-MCCs (Fig. 5D, yellow circle) and newly born, amplified centrioles in MCCs (Fig. 5D, yellow boxes). In the early stages of MCC differentiation, the CEP192^AID^ signal clustered around the centrioles and decreased as cells completed differentiation with motile cilia (Fig. 5D, yellow box no. 4). After 1 hour of 5-Ph-IAA treatment, CEP192^AID^ was nearly undetectable in MCCs and non-MCCs in the presence of *OsTir1* and stayed low over the entire observation period of 48 hours (Fig. 5, D to F, and fig. S5D).

Given that CEP192 localizes to centrioles during MCC differentiation, we next tested whether CEP192 was required for centriole amplification and motile cilia formation. *Cep192^AID/AID^* mTECs with or without *OsTir1* were cultured at ALI for 7 days in the presence of 5-Ph-IAA. We observed no difference in the number of MCCs (FOXJ1+ cells) or cells with motile cilia (Fig. 5, G to I). We also performed these experiments in the presence of verapamil, an efflux pump inhibitor, as some drugs are ineffective in mTECs due to the efflux capability of these epithelial cells (*43*). 5-Ph-IAA effectiveness was not affected by verapamil (Fig. 5, G and H), suggesting that this compound is not sensitive to efflux pumps. To assess whether CEP192 is required for cilia maintenance, we allowed the cultures to differentiate for 7 days at ALI and then treated with 5-Ph-IAA for 2 days (ALI days 7 to 9). The number of FOXJ1+ cells and cells bearing motile cilia was similar in untreated cells and 5-Ph-IAA–treated cells, showing that CEP192 is dispensable for cilia maintenance (fig. S5, E to G). Together, we conclude that CEP192 function is not required for differentiation, centriole amplification, or motile cilia formation and maintenance in MCCs.

### Long-term CEP192 depletion causes weight loss, mitotic errors, and cell death in the intestine

**Fig. 6.**
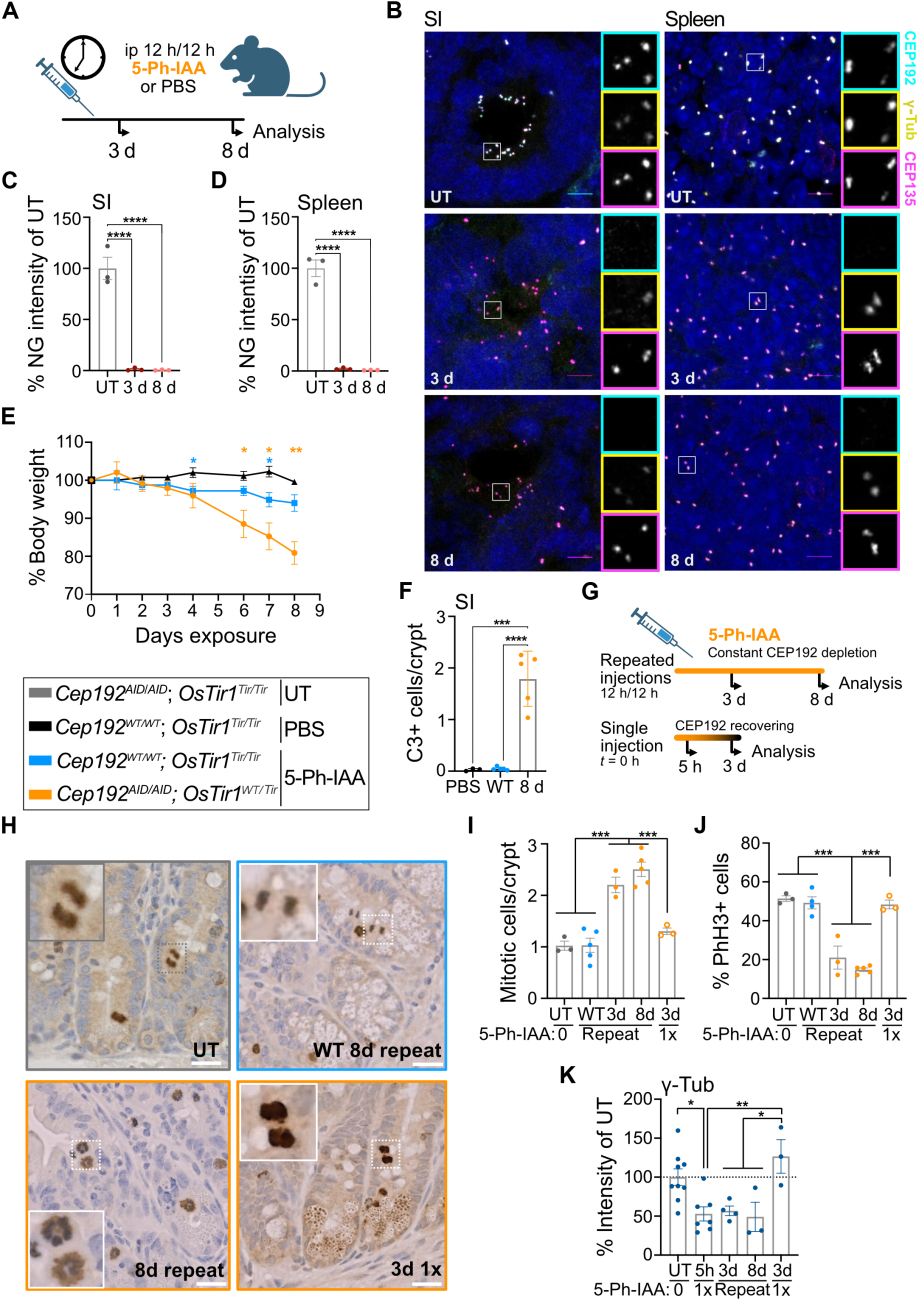
Chronic degradation of CEP192^AID^ causes mitotic failure and cell death in the gastrointestinal tract. (**A**) Mice were injected with PBS or 5-Ph-IAA every 12 hours. (**B**) Representative images of the SI and the spleen of *Cep192^AID/AID^*; *OsTir1*^WT/*Tir*^ mice left untreated (UT) or injected with 5-Ph-IAA. Scale bars, 5 μm. (**C** and **D**) Graphs showing the CEP192^AID^ signal [NeonGreen (NG)] in images of the (C) SI and the (D) spleen. (**E**) Body weight of *Cep192^AID/AID^*; *OsTir1*^WT/*Tir*^ mice repeatedly injected with 5-Ph-IAA. *N* = 5 mice per genotype (8 days); *N* = 3 mice per genotype (3 days). (**F**) Quantification of cell death measured by cleaved caspase-3 (C3) in the crypts of the SI. *N* = 3 to 5 mice per genotype and treatment; *n* = 100 to 150 crypts per mouse. (**G**) Mice were left untreated or treated for 3 or 8 days with 5-Ph-IAA. To study recovery, mice injected once were analyzed 5 or 72 hours after 5-Ph-IAA administration. (**H** to **J**) Number and morphology of mitotic cells in the SI of *Cep192^AID/AID^* mice that were wild type or heterozygous for *OsTir1*. Mice were left untreated (UT), treated with 5-Ph-IAA for 3 days (3d) and 8 days (8d), or injected once and analyzed after 3 days (3d 1x). *N* = 3 to 5 mice per condition. (H) Representative images of phH3-stained tissues. Insets highlight mitotic cells. Scale bars, 20 μm. (I) Graph showing the number of mitotic cells per crypt. *n* = 100 to 250 crypts per mouse. (J) Quantification of the percentage of phH3+ cells with metaphase or anaphase-like morphology. *n* = 100 to 300 cells per mouse. (**K**) Graph showing the signal intensity of γ-tubulin in *Cep192^AID/AID^*; *OsTir1*^WT/*Tir*^ mice injected with 5-Ph-IAA once (5h 1x; 3d 1x) or repeatedly (8d; 3d). *N* = 3 to 9 mice per condition; *n* = 50 to 100 cells per mouse. Data are shown as means ± SEM. Statistical significance was measured using one-way ANOVA [(C), (D), and (F)] or two-way ANOVA [(I) to (K)] with Sidak’s multiple comparisons test. Only significant results are indicated. ns *P* ≥ 0.05; *P < 0.05; **P < 0.01; ***P < 0.001; ****P < 0.0001.

We used the AID2 system to investigate the long-term effects of depletion of CEP192 in mice. Because we observed minimal recovery of CEP192^AID^ after 24 hours (Fig. 3F), we injected *Cep192^AID/AID^*; *OsTir1^WT/Tir^* mice every 12 hours for 3 or 8 days with 5-Ph-IAA or PBS (Fig. 6A). After 3 days, the CEP192^AID^ protein level at the centrosome was reduced by >95% in the spleen, SI, and other tissues analyzed (Fig. 6, B to D, and fig. S6, A to C). As expected, repeated dosing with 5-Ph-IAA was well tolerated in control mice, with minimal weight loss. By contrast, *Cep192^AID/AID^*; *OsTir1^WT/Tir^* mice lost up to 20% body weight (humane endpoint) after 8 days of 5-Ph-IAA treatment (Fig. 6E). These animals displayed gastrointestinal symptoms and massive cell death in crypts of the SI and the colon, as indicated by active caspase-3 staining (Fig. 6F and fig. S6, D to H). Although the level of cell death was not significantly elevated in the spleen following CEP192^AID^ loss (fig. S6, I and J), the spleens of *Cep192^AID/AID^*; *OsTir1^WT/Tir^* mice were markedly smaller after 8 days of 5-Ph-IAA treatment (fig. S6K). Sustained CEP192^AID^ degradation resulted in an ~2.5-fold increased mitotic index in the intestinal crypts (Fig. 6, G to I), and most of the mitotic figures had a monopolar morphology (Fig. 6, H and J). Similar to the findings in MEFs (Fig. 4, I to L), acute (5 hours) and sustained (3 and 8 days) CEP192^AID^ loss reduced centrosomal γ-tubulin by ~50% in the SI (Fig. 6, B and K). The mitotic index, centrosomal γ-tubulin, and the presence of bipolar spindles were restored when CEP192^AID^ levels had fully recovered at 72 hours after a single 5-Ph-IAA injection (Figs. 3, C and E, and 6, G to K). Together, these data show that repeated dosing with 5-Ph-IAA results in the degradation of >95% of centrosomal CEP192^AID^ in vivo, leading to mitotic delays and cell death in proliferating intestinal cells.

## DISCUSSION

Here, we show that the second-generation AID system is well suited for acute and long-term targeted protein degradation in live mice. The AID2 system has been used successfully in *C. elegans* and *D. melanogaster*, and a proof-of-principle study by Yesbolatova *et al.* showed that the AID2 system effectively degraded a fluorescent reporter in mice (*18*, *44*, *45*). Our data add to this by showing that the AID2 system can drive near-complete degradation of an essential, endogenously tagged protein in live mice. The first-generation AID system uses IAA, which is toxic in vivo (Fig. 1), limiting its applications to ex vivo studies with primary cells or cell lines (*10*, *19*). In contrast, 5-Ph-IAA is well tolerated at concentrations required for protein degradation, and we did not observe leaky degradation in the absence of 5-Ph-IAA, as described with the first-generation AID system (Fig. 1) (*42*).

Other systems for in vivo protein degradation in mice, namely, dTAG and PROTACs, depend on large, complex molecules. Previous studies showed that effective delivery of these compounds requires vehicle formulations that can cause toxicity or inflammation, particularly after repeated injections (*7*, *11*). An advantage of AID2 over these systems is that 5-Ph-IAA is a small molecule molecular glue that can be administered in PBS or saline. In contrast to the dTAG or the first-generation AID system, we did not observe any toxicity or inflammation at the injection site for 5-Ph-IAA (*11*, *19*). Moreover, we show that repeated dosing over 2 weeks with a concentration of 5-Ph-IAA that induces efficient degradation does not affect the well-being of the animals (Figs. 2 and 6). Besides toxicity, another advantage of the AID2 system is the remarkably fast degradation dynamics (Fig. 3) (*7*, *9*, *10*, *18*). Our in vivo time course shows that, depending on the tissue, ~60 to 90% degradation can be achieved within 30 min and >95% degradation within 5 hours. This makes this system suitable for studying fast processes such as mitosis or cell death. One limitation of the AID2 system compared to the dTAG system is the requirement for more complex mouse genetics. Although both the AID2 and dTAG rely on endogenous tagging of the gene of interest, the AID2 system also requires the expression of *OsTir1*. The conditional *OsTir1* allele, however, allows for tissue-specific degradation, which is difficult to achieve with other approaches.

**Fig. 7.**
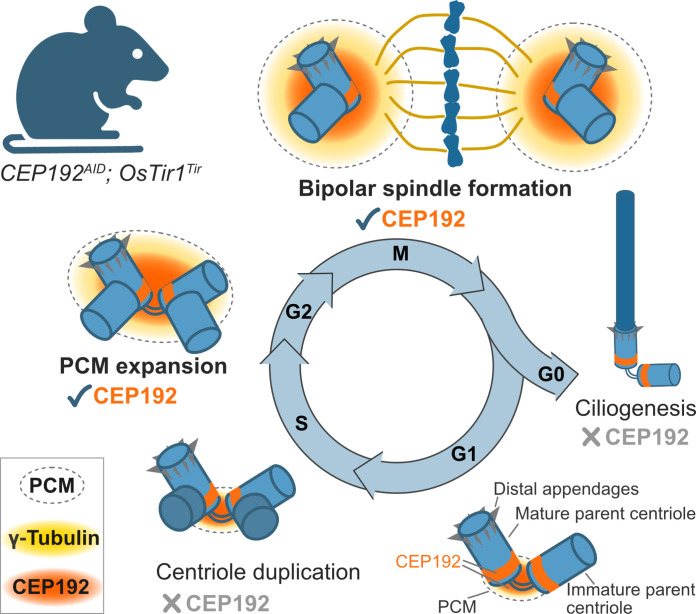
Murine CEP192 is required for bipolar spindle assembly in mitosis but not centriole duplication or ciliogenesis. Model summarizing the functions of CEP192 investigated in mouse tissues, ex vivo cultures, and MEFs. Mouse CEP192 is essential for recruiting γ-tubulin to form a bipolar spindle in mitosis. Albeit its presence on centrioles, CEP192 is not required for centriole duplication in mice. Mouse CEP192 localizes to basal bodies but is dispensable for the formation and maintenance of primary and motile cilia.

The DepMap classifies CEP192 as a common-essential protein, and therefore, it is not possible to study its function in mice in vivo with knockout mouse models. Here, we explored CEP192 biology in different cell types using the AID2 system in primary cultures and live mice. Previous studies using cultured human cell lines have relied on RNA interference for reducing CEP192 levels and reported contradictory results on whether CEP192 is required for centriole duplication (*28*–*31*, *35*, *36*). We found that murine CEP192 is essential for successful mitosis, whereas centriole duplication, integrity, and maturation were not affected by CEP192 loss in MEFs (Figs. 4 and 7). Our data support prior works suggesting that the main function of CEP192 is in recruiting γ-tubulin for microtubule nucleation and bipolar spindle formation (*31*, *35*–*37*). Following CEP192 degradation, MEFs formed monopolar spindles, aborted mitosis, and became polyploid. Proliferating intestinal cells also required CEP192 for successful mitosis (Fig. 6). Degradation of CEP192 for 8 days caused gastrointestinal defects because of massive cell death in the crypts of the small and large intestines. CEP192 depletion reduced the abundance of γ-tubulin in the SI, and most mitotic cells showed a monopolar spindle morphology. Together with the increased mitotic index, this indicates that, in the absence of CEP192, proliferating intestinal cells cannot complete mitosis and undergo cell death. Moreover, continuous depletion of CEP192 reduced the size of the spleen, suggesting that cells in this proliferative organ also die in the absence of CEP192, presumably due to errors in mitosis (fig. S6). Last, we addressed the unstudied role of CEP192 in the formation of primary and motile cilia (Fig. 5). Although CEP192 is found at centrosomes and newly born centrioles in MEFs and MCCs, we show that it is dispensable for ciliogenesis and cilia maintenance.

In summary, our data indicate that the essential in vivo role of CEP192 is in mitotic spindle assembly, explaining why proliferating tissues exhibit a high dependence on CEP192 (Fig. 7). The *OsTir1* allele we created is conditional, allowing expression to be driven by tissue-specific *Cre* activity. Conditional expression of *OsTir1* in less proliferative tissues is likely to overcome the limited tolerance to whole-body depletion of CEP192 and enable future investigation of cell type–specific functions of CEP192.

## MATERIALS AND METHODS

### Experimental design

We created mouse models expressing OSTIR1-F74G and endogenously tagged *Cep192* to characterize the tolerance and efficacy of the AID2 system across tissues and over time in ex vivo cultures and live mice.

### Mouse models

*Rosa26^LSL-OSTir1-F74G^* and *Cep192*^mAID-mNeonGreen^ mice were generated using CRISPR-Cas9 technology. Briefly, Cas9 protein (30 ng/μl, PNABio), tracrRNA (0.6 μM, Dharmacon), crRNA (0.6 μM, IDT), and ssDNA (single-stranded DNA) oligonucleotide (10 ng/μl, IDT) were mixed and diluted in ribonuclease-free injection buffer [10 mM tris-HCl (pH 7.4) and 0.25 mM EDTA] and injected into the pronucleus of one-cell embryos and subsequently transplanted into pseudopregnant ICR females. The single guide RNAs (sgRNAs) were chosen based on the location of the available protospacer adjacent motif (PAM) sites and a minimal number of predicted off-target sites according to crispor.tefor.net. Injections and transplantation were performed by the JHU Transgenic Core.

*Rosa26^LSL-OsTir1-F74G^* mice were generated by targeting the *Rosa26* locus as described before (*46*). The sgRNA and the targeting vector containing a lox-stop-lox cassette, codon-optimized *OsTir1* (*47*) harboring the F74G modification, and a Myc tag (Fig. 1C) were coinjected into B6SJL/F2 embryos.

sgRNA: 5′-actccagtctttctagaaga

Primers used for genotyping and sequencing to characterize the offspring:

ROSA-P1: 5′-AAA GTC GCT CTG AGT TGT TAT

ROSA-P2: 5′-GCG AAG AGT TTG TCC TCA ACC

ROSA-P3: 5′-GGA GCG GGA GAA ATG GAT ATG

Following one round of backcross to a C57B/6J wild type, *Rosa26^LSL-OsTir1-F74G^* were crossed to Sox2-Cre females (*48*) (the Jackson Laboratory strain no. 008454) to achieve full body expression of OSTIR1 by excision of the lox-Stop-lox cassette (*Rosa26^OsTir1-F74G^*).

*Cep192*^mAID-mNeonGreen^ mice were generated by coinjection of an sgRNA and a DNA donor construct to target the last exon (48) of *Cep192* (Fig. 1E). Offspring with correct insertion was crossed to C57BL/6J wild type for two generations and then intercrossed to generate homozygous animals.

sgRNA1: 5′-cgactaatcggtgaatccct

DNA donor template: 5′-AGATGAAGGCAAGAGTATTGCTATTCGACTAATCGGCGAGTCTCTTGGAAAAAGTGGAGGTGCTGGCTCTGGACCTGGCTCTGGAGCAGGCGGTGGCAGCGGGGATCCAGCAGACTCTAAAGAGAAGTCAGCTTGTCCTAAGGACCCTGCCAAGCCACCTGCGAAGGCCCAAGTAGTCGGTTGGCCACCCGTGCGCAGCTATAGGAAGAACGTGATGGTGTCCTGTCAGAAGTCTAGCGGGGGGCCCGAGGCTAGCGGCGGATCTTCCTCAAAGGGAGAAGAAGACAACATGGCCTCACTGCCCGCAACACACGAGCTGCATATTTTCGGAAGCATCAATGGGGTGGATTTCGACATGGTGGGCCAGGGGACTGGAAACCCAAATGATGGGTACGAGGAACTGAATCTGAAGTCAACCAAAGGCGACCTCCAGTTCAGCCCTTGGATTCTGGTGCCCCACATTGGCTATGGGTTTCATCAGTATCTGCCCTACCCTGACGGAATGTCCCCATTCCAGGCAGCTATGGTGGATGGATCTGGCTACCAGGTCCACAGGACCATGCAGTTTGAGGACGGGGCCAGTCTGACTGTGAACTACCGCTATACCTACGAGGGATCACATATCAAGGGCGAAGCACAGGTGAAAGGGACAGGATTCCCAGCTGATGGCCCCGTCATGACAAACTCTCTGACCGCCGCCGACTGGAGCCGGTCCAAGAAAACTTACCCTAACGATAAGACCATCATCTCTACCTTCAAGTGGAGTTATACCACAGGCAACGGGAAGCGGTACAGAAGCACAGCCCGAACTACCTATACTTTTGCTAAGCCCATGGCTGCAAACTATCTGAAAAATCAGCCTATGTACGTGTTCAGAAAGACCGAGCTGAAGCACTCCAAAACAGAACTGAATTTCAAGGAATGGCAGAAGGCTTTTACCGATGTGATGGGGATGGACGAACTGTATAAGTAACTAGAATACATATTGTGTAAATGACCTGCTTATAT.

Primers used for genotyping and allele characterization:

F1: 5′-GGT GCA CTT CAG ACC AGA GG

R2: 5′-TGT AGA AAA ATG AGA TTG CCA AA

AID-R: 5′-TGA CAG GAC ACC ATC ACG TT

*Rosa26^LSL-OsTir1-F74G^* (JAX stock no. 039572) and *Cep192*^mAID-mNeonGreen^ (JAX stock no. 039573) mice are available from the Jackson Laboratory.

Mice were housed under standard conditions in an AAALAC-accredited facility. All animal experiments were approved by the Johns Hopkins University Animal Care and Use Committee (MO21M300). Age-matched 6- to 15-week-old mice were used in a balanced sex ratio in all experiments.

### 5-Ph-IAA treatment in vivo

For intraperitoneal injections, 5-phenyl-1*H*-indole-3-acetic acid (5-Ph-IAA; no. 30-003, BioAcademia, Osaka, Japan) was dissolved in sterile PBS. Specifically, 10 mg of 5-Ph-IAA was dissolved in 1 ml of PBS under dropwise addition of NaOH. Once dissolved, the pH was adjusted to 7.4 using minute amounts of HCl and reconstituted with PBS to achieve a concentration of 1 mg/ml. The solution was sterile filtered using 0.2-μm syringe filters. Mice were injected intraperitoneally with 1 or 5 mg/kg either once or repeatedly every 24 hours (Figs. 2 and 3) or every 12 hours (Fig. 6). Control mice were injected with PBS for repeat dose experiments or left untreated for single dosing.

### Blood and serum analysis

Complete blood cell counts were performed by the Johns Hopkins University Phenotyping and Pathology Core using a DEXX ProCyte Dx Hematology Analyzer. The standard tox panel of serum parameters was analyzed by IDEXX BioAnalytics (North Grafton, MA, USA).

### Histopathological assessment

Hematoxylin and eosin–stained sections (4 μm) of paraffin-embedded tissue were used for histopathological assessment by a board-certified veterinary pathologist at the Johns Hopkins University Phenotyping and Pathology Core.

### Immunohistochemistry

Sections (4 μm) of paraffin-embedded SI tissue were deparaffinized and rehydrated. Following antigen retrieval in sodium citrate buffer using a steamer, the tissue sections were blocked in 3% hydrogen peroxidase solution containing Tween 20 and goat serum. The samples were incubated with primary antibody [Phospho-histone H3 (Ser^10^), rabbit polyclonal, Cell Signaling, no. 9701; Cleaved Caspase-3 (Asp^175^), rabbit polyclonal, Cell Signaling, no. 9661] overnight. After washing steps and incubation with the secondary antibody [Biotin-SP-conjugated AffiniPure Goat anti-Rabbit immunoglobulin G (IgG) (H+L), Jackson ImmunoResearch Laboratories, no. 111-065-144], the signal was amplified (VECTASTAIN Elite ABC-HRP kit, Vector Laboratories, no. PK-6100) and developed (ImmPACT DAB Peroxidase Substrate, Vector Laboratories, no. SK-4105). The tissue sections were imaged on an Axioscan 6 Slidescanner (Zeiss).

### Isolation and differentiation of mTECs

mTECs were isolated, cultured, and differentiated as previously described (*49*). In brief, tracheas were dissected and incubated in Pronase (Roche) at 4°C overnight. Following enzymatic and mechanical dissociation, the tracheal cells were seeded onto 0.4-μm Falcon transwell membranes (Corning). After 5 days of proliferation, the medium was removed from the apical chamber and the basal medium was changed to NuSerum medium, starting the ALI culture (ALI day 0). Cells were allowed to differentiate up to 9 days. For experiments in Fig. 5 (E and G to I), 1 μM 5-Ph-IAA was added on ALI day 0. To assess cilia maintenance, the mTEC cultures were treated with 1 μM 5-Ph-IAA on days 7 to 9 (fig. S5, E to G). For the time course experiments, 1 μM 5-Ph-IAA was added on ALI day 5 (Fig. 5F and fig. S5D). The basal medium with or without 5-Ph-IAA was refreshed every 1 to 2 days.

### Generation and culture of MEFs

E12.5-14.5 embryos were isolated as previously described (*50*). In brief, the embryo bodies were digested at 4°C overnight in 0.05% Trypsin-EDTA (Gibco, 25300062). After 5-min incubation at 37°C, the MEF cells were dissociated by pipetting. MEFs were cultured in Dulbecco’s modified Eagle’s medium (Corning, no. 10-017-CV) containing 10% fetal bovine serum (FBS) (Corning, no. 35-010-CV), penicillin, streptomycin (Gibco, no. 10378016), and 0.1 mM β-mercaptoethanol at 37°C in 5% CO_2_ and 3% O_2_ atmosphere. For immortalization, MEFs were exposed to the SV40 T antigen and passaged for 3 weeks before performing experiments. To induce degradation, the MEFs were treated with 1 μM 5-Ph-IAA in dimethyl sulfoxide (DMSO) or with DMSO as control for the indicated time periods. For MEF ciliation, media were removed, cells were washed with PBS, and then serum starvation media (normal MEF media except only 0.5% FBS) were added either with or without 1 μM 5-Ph-IAA. To dilute out centrioles, MEFs were treated with 500 nM centrinone (MedChemExpress, no. HY-18682) for up to 72 hours.

### Imaging

Immunofluorescence: Fresh tissue was embedded in TissueTek O.C.T. compound (Sakura Finetek), sectioned on a Leica CM1950 cryostat (20 μm) and collected on Superfrost Plus microscope slides (Thermo Fisher Scientific). To examine ciliogenesis, MEFs and mTECs on coverslips were fixed in 4% paraformaldehyde (Electron Microscopy Sciences, no. 15714) in PBS. Sectioned tissues and MEFs on coverslips for all other experiments were fixed first in 1.5% paraformaldehyde and then in −20°C cold methanol for 4 min each. Following blocking in 2.5% FBS, 200 mM glycine, and 0.1% Triton X-100 in PBS for 1 hour, the samples were incubated with the respective primary antibodies in the same buffer for 1 hour. The samples were washed three times with PBS + 0.5% Triton X-100 and incubated with the secondary antibodies (Invitrogen) and DAPI (4′,6-diamidino-2-phenylindole). Samples were washed three times and mounted in Prolong Gold Antifade (Life Technologies, no. P36930). Primary antibodies used were CEP135 (rabbit polyclonal, homemade, Alexa555-conjugated, 1:500), γ-tubulin (goat polyclonal, homemade, Alexa647-conjugated, 1:500), CEP152 (rabbit polyclonal, homemade, Alexa488-conjugated, 1:500), PLK4 (rabbit polyclonal, homemade, Alexa555-conjugated, 1:500), CEP164 (rabbit polyclonal, EMD Millipore Corp., ABE2621, 1:1000), α-tubulin (guinea pig monoclonal, Sysy Antibodies, no. 302 308, 1:1000) CEP192 (rabbit polyclonal, gift from K. Oegema, 1:1000), FOXJ1 (mouse monoclonal, Thermo Fisher Scientific, no. 14-9965-82, 1:1000), acetyl-α-tubulin [rabbit polyclonal, Lys^40^ (D20G3), Cell Signaling, no. 5335T, 1:1000], mouse anti-PCNT (1:250, BD Transduction Laboratories, no. 611814), and ARL13B (mouse monoclonal, N295B66, Antibodies Incorporated, no. 75-287, 1:1000). Because the CEP152 antibody used was directly conjugated to Alexa488 and thus interfering with the CEP192-NeonGreen signal, CEP152 was quantified using an anti-rabbit Alexa555-conjugated secondary antibody. Tissue sections and MEFs (primary cilia assessment) were imaged on an SP8 confocal microscope (Leica Microsystems) using a Leica 40× 1.30–numerical aperture (NA) oil objective at 0.2- to 0.5-μm *z*-sections. For degradation analysis, MEFs were imaged using a Deltavision Elite system (GE Healthcare) or on a Thunder Imager (Leica Microsystems) with an Olympus 63× 1.42-NA oil objective at 0.2-μm *z*-sections. mTECs were imaged on a Zeiss Axio Observer 7 inverted microscope with Slidebook 2023 software (3i—Intelligent, Imaging Innovations Inc.), CSU-W1 (Yokogawa) T1 Super-Resolution Spinning Disk, and Prime 95B CMOS camera (Teledyne Photometrics) with a 63x plan-apochromat oil immersion objective with 1.4 NA.

Live imaging: MEFs were seeded in separate 4-well Cellview cell culture dishes (Greiner) and live cell imaged on an SP8 confocal microscope at 37°C, 5% CO_2_. 5-Ph-IAA (1 μM) was added to at *t* = 0 min, and images were taken of every 5 min for 75 min using a Leica 63×, 1.40-NA oil objective with 0.5-μm *z*-sections.

To measure mitotic duration and fate, MEFs were imaged (bright field) using an IncuCyte S3 (Sartorius) every 20 min for up to 48 hours. To induce degradation, 5-Ph-IAA (1 μM) was added at *t* = 0 min. The time in the stills shown in fig. S4C was adjusted so that one frame before rounding up of the cell was set to a time point of 0 min.

### Image analysis

All imaging analysis was performed blinded using ImageJ (v2.1.0/1.53c, US National Institutes of Health, http://imagej.net). For tissue sections, immunofluorescence images were lightning processed using LAS X Software (Leica, v3.5.6.21594) and maximum intensity projections (16 bit) were generated using ImageJ. Centrioles of MEFs were assessed on deconvolved two-dimensional maximum intensity projections (16 bit). For live imaging of CEP192^AID^ degradation in MEFs, the CEP192^AID^ (NeonGreen) signal was quantified per frame in maximum intensity projected movies. For fixed and live imaging, signal intensities of CEP192^AID^ (NeonGreen), CEP135, and γ-tubulin were determined by drawing a circular region of interest (ROI_S_) around the centrosome and a larger circular ROI (ROI_L_) around the ROI_S_. The signal in ROI_S_ was calculated using the formula *I*_S_ − [(*I*_L_ − *I*_S_)/(*A*_L_ − *A*_S_) × *A*_S_], where *A* is the area and *I* is the integrated intensity.

For IncuCyte live imaging, mitotic cells were followed over time to determine the fate and the duration of mitosis. The beginning of mitosis was defined as rounding up of the cell. The end of mitosis was given by one of three possible fates: (i) successful division with a visible metaphase plate or anaphase and readhering of two daughter cells, (ii) readhering without division (abortive mitosis), or (iii) cell death.

### Protein extraction and immunoblotting

Snap-frozen tissues were homogenized in radioimmunoprecipitation assay lysis buffer (150 mM NaCl, 50 mM Tris, 1% NP-40, 0.5% sodium deoxycholate, 1% SDS, and one tablet of EDTA-free protease inhibitors), and protein content was determined using a Bradford protein assay (Bio-Rad, no. 5000001). Fifty to 100 μg of protein was run on an SDS–polyacrylamide gel electrophoresis and blotted using a wet-transfer system (Bio-Rad) to a nitrocellulose membrane (0.45 μm, Santa Cruz Biotechnology, no. sc-3724). Transfer quality and total protein were assessed by Ponceau S staining before the membranes were probed for Myc tag (Abcam, no. ab9106, 1:1000). Fluorophore-conjugated secondary antibodies were used for detection using the LI-COR Odyssey CLx system (anti-rabbit IgG, Thermo Fisher Scientific, SA5-35571, 1:10,000).

### Flow cytometry

Following fixation of MEF cells in 70% EtOH, DNA was stained with propidium iodide (30 μg/ml). Ploidy distribution was assessed on an LSR Fortessa (BD Biosciences), and data were analyzed using FlowJo software (v10).

### Statistical analysis

Statistical analysis was performed using GraphPad Prism (v9.0.0, GraphPad Software LLC). Two-tailed, unpaired Student’s *t* test was used for comparison of two groups. One-way analysis of variance (ANOVA) or two-way ANOVA with Sidak’s or Dunnett’s multiple comparisons test was used for three or more groups. Significance levels and tests performed are stated in the figure legends; *P < 0.05, **P < 0.01, ***P < 0.001, and ****P < 0.0001. In some graphs, only statistically significant results are indicated. “*N*” represents the number of animals, and “*n*” refers to the number of cells analyzed per mouse. Each mouse is considered a biological replicate.

## References

[1] D. P. Bondeson, A. Mares, I. E. D. Smith, E. Ko, S. Campos, A. H. Miah, K. E. Mulholland, N. Routly, D. L. Buckley, J. L. Gustafson, N. Zinn, P. Grandi, S. Shimamura, G. Bergamini, M. Faelth-Savitski, M. Bantscheff, C. Cox, D. A. Gordon, R. R. Willard, J. J. Flanagan, L. N. Casillas, B. J. Votta, W. den Besten, K. Famm, L. Kruidenier, P. S. Carter, J. D. Harling, I. Churcher, C. M. Crews, Catalytic in vivo protein knockdown by small-molecule PROTACs. Nat. Chem. Biol. 11, 611–617 (2015). 26075522 10.1038/nchembio.1858 PMC4629852

[2] R. Verma, D. Mohl, R. J. Deshaies, Harnessing the power of proteolysis for targeted protein inactivation. Mol. Cell 77, 446–460 (2020). 32004468 10.1016/j.molcel.2020.01.010

[3] T. Wu, H. Yoon, Y. Xiong, S. E. Dixon-Clarke, R. P. Nowak, E. S. Fischer, Targeted protein degradation as a powerful research tool in basic biology and drug target discovery. Nat. Struct. Mol. Biol. 27, 605–614 (2020). 32541897 10.1038/s41594-020-0438-0 PMC7923177

[4] C. Grohmann, C. M. Magtoto, J. R. Walker, N. K. Chua, A. Gabrielyan, M. Hall, S. A. Cobbold, S. Mieruszynski, M. Brzozowski, D. S. Simpson, H. Dong, B. Dorizzi, A. V. Jacobsen, E. Morrish, N. Silke, J. M. Murphy, J. K. Heath, A. Testa, C. Maniaci, A. Ciulli, G. Lessene, J. Silke, R. Feltham, Development of NanoLuc-targeting protein degraders and a universal reporter system to benchmark tag-targeted degradation platforms. Nat. Commun. 13, 2073 (2022). 35440107 10.1038/s41467-022-29670-1 PMC9019100

[5] K. M. Sakamoto, K. B. Kim, A. Kumagai, F. Mercurio, C. M. Crews, R. J. Deshaies, Protacs: Chimeric molecules that target proteins to the Skp1–Cullin–F box complex for ubiquitination and degradation. Proc. Natl. Acad. Sci. U.S.A. 98, 8554–8559 (2001). 11438690 10.1073/pnas.141230798 PMC37474

[6] M. Békés, D. R. Langley, C. M. Crews, PROTAC targeted protein degraders: The past is prologue. Nat. Rev. Drug Discov. 21, 181–200 (2022). 35042991 10.1038/s41573-021-00371-6 PMC8765495

[7] B. Nabet, J. M. Roberts, D. L. Buckley, J. Paulk, S. Dastjerdi, A. Yang, A. L. Leggett, M. A. Erb, M. A. Lawlor, A. Souza, T. G. Scott, S. Vittori, J. A. Perry, J. Qi, G. E. Winter, K.-K. Wong, N. S. Gray, J. E. Bradner, The dTAG system for immediate and target-specific protein degradation. Nat. Chem. Biol. 14, 431–441 (2018). 29581585 10.1038/s41589-018-0021-8 PMC6295913

[8] X. Sun, J. Wang, X. Yao, W. Zheng, Y. Mao, T. Lan, L. Wang, Y. Sun, X. Zhang, Q. Zhao, J. Zhao, R.-P. Xiao, X. Zhang, G. Ji, Y. Rao, A chemical approach for global protein knockdown from mice to non-human primates. Cell Discov. 5, 10 (2019). 30729032 10.1038/s41421-018-0079-1 PMC6361926

[9] B. Nabet, F. M. Ferguson, B. K. A. Seong, M. Kuljanin, A. L. Leggett, M. L. Mohardt, A. Robichaud, A. S. Conway, D. L. Buckley, J. D. Mancias, J. E. Bradner, K. Stegmaier, N. S. Gray, Rapid and direct control of target protein levels with VHL-recruiting dTAG molecules. Nat. Commun. 11, 4687 (2020). 32948771 10.1038/s41467-020-18377-w PMC7501296

[10] A. Abuhashem, A. S. Lee, A. L. Joyner, A. K. Hadjantonakis, Rapid and efficient degradation of endogenous proteins in vivo identifies stage-specific roles of RNA Pol II pausing in mammalian development. Dev. Cell 57, 1068–1080.e6 (2022). 35421370 10.1016/j.devcel.2022.03.013 PMC9047393

[11] P. Yenerall, T. Sung, K. Palyada, J. Qian, S. Arat, S. W. Kumpf, S.-W. Wang, K. Biddle, C. Esparza, S. Chang, W. Scott, W. Collette, T.-S. Winrow, T. Affolter, N. Shirai, S. Thibault, J. Wang, L. Liu, M. Bauchmann, J. Frey, S. Steyn, A. Sacaan, A. Vitsky, Y. Ahn, T. Paul, L. Lum, J. Oyer, A. Yang, W. Hu, Use of the dTAG system in vivo to degrade CDK2 and CDK5 in adult mice and explore potential safety liabilities. Toxicol. Sci. 194, 53–69 (2023). 37228089 10.1093/toxsci/kfad049 PMC10306401

[12] M. Pettersson, C. M. Crews, PROteolysis TArgeting Chimeras (PROTACs)—Past, present and future. Drug Discov. Today Technol. 31, 15–27 (2019). 31200855 10.1016/j.ddtec.2019.01.002 PMC6578591

[13] K. Nishimura, T. Fukagawa, H. Takisawa, T. Kakimoto, M. Kanemaki, An auxin-based degron system for the rapid depletion of proteins in nonplant cells. Nat. Methods 6, 917–922 (2009). 19915560 10.1038/nmeth.1401

[14] A. J. Holland, D. Fachinetti, J. S. Han, D. W. Cleveland, Inducible, reversible system for the rapid and complete degradation of proteins in mammalian cells. Proc. Natl. Acad. Sci. U.S.A. 109, E3350–E3357 (2012). 23150568 10.1073/pnas.1216880109 PMC3523849

[15] K. Nishimura, R. Yamada, S. Hagihara, R. Iwasaki, N. Uchida, T. Kamura, K. Takahashi, K. U. Torii, T. Fukagawa, A super-sensitive auxin-inducible degron system with an engineered auxin-TIR1 pair. Nucleic Acids Res. 48, e108 (2020). 32941625 10.1093/nar/gkaa748 PMC7544234

[16] A. N. Goldner, S. M. Fessehaye, N. Rodriguez, K. A. Mapes, M. Osterfield, K. Doubrovinski, Evidence that tissue recoil in the early *Drosophila* embryo is a passive not active process. Mol. Biol. Cell 34, br16 (2023). 37405768 10.1091/mbc.E22-09-0409 PMC10551697

[17] T. Negishi, S. Kitagawa, N. Horii, Y. Tanaka, N. Haruta, A. Sugimoto, H. Sawa, K.-I. Hayashi, M. Harata, M. T. Kanemaki, The auxin-inducible degron 2 (AID2) system enables controlled protein knockdown during embryogenesis and development in Caenorhabditis elegans. Genetics 220, iyab218 (2022). 34865044 10.1093/genetics/iyab218 PMC9208642

[18] A. Yesbolatova, Y. Saito, N. Kitamoto, H. Makino-Itou, R. Ajima, R. Nakano, H. Nakaoka, K. Fukui, K. Gamo, Y. Tominari, H. Takeuchi, Y. Saga, K.-I. Hayashi, M. T. Kanemaki, The auxin-inducible degron 2 technology provides sharp degradation control in yeast, mammalian cells, and mice. Nat. Commun. 11, 5701 (2020). 33177522 10.1038/s41467-020-19532-z PMC7659001

[19] L. Macdonald, G. C. Taylor, J. M. Brisbane, E. Christodoulou, L. Scott, A. von Kriegsheim, J. Rossant, B. Gu, A. J. Wood, Rapid and specific degradation of endogenous proteins in mouse models using auxin-inducible degrons. eLife 11, e77987 (2022). 35736539 10.7554/eLife.77987 PMC9273210

[20] J. M. Suski, N. Ratnayeke, M. Braun, T. Zhang, V. Strmiska, W. Michowski, G. Can, A. Simoneau, K. Snioch, M. Cup, C. M. Sullivan, X. Wu, J. Nowacka, T. B. Branigan, L. R. Pack, J. A. DeCaprio, Y. Geng, L. Zou, S. P. Gygi, J. C. Walter, T. Meyer, P. Sicinski, CDC7-independent G1/S transition revealed by targeted protein degradation. Nature 605, 357–365 (2022). 35508654 10.1038/s41586-022-04698-x PMC9106935

[21] B. G. Lambrus, T. C. Moyer, A. J. Holland, Applying the auxin-inducible degradation system for rapid protein depletion in mammalian cells. Methods Cell Biol. 144, 107–135 (2018). 29804665 10.1016/bs.mcb.2018.03.004

[22] S. Yoshiba, Y. Tsuchiya, M. Ohta, A. Gupta, G. Shiratsuchi, Y. Nozaki, T. Ashikawa, T. Fujiwara, T. Natsume, M. T. Kanemaki, D. Kitagawa, HsSAS-6-dependent cartwheel assembly ensures stabilization of centriole intermediates. J. Cell Sci. 132, jcs217521 (2019). 31164447 10.1242/jcs.217521

[23] A. Vásquez-Limeta, K. Lukasik, D. Kong, C. Sullenberger, D. Luvsanjav, N. Sahabandu, R. Chari, J. Loncarek, CPAP insufficiency leads to incomplete centrioles that duplicate but fragment. J. Cell Biol. 221, e202108018 (2022). 35404385 10.1083/jcb.202108018 PMC9007748

[24] M. van Toorn, A. Gooch, S. Boerner, T. Kiyomitsu, NuMA deficiency causes micronuclei via checkpoint-insensitive k-fiber minus-end detachment from mitotic spindle poles. Curr. Biol. 33, 572–580.e2 (2023). 36626904 10.1016/j.cub.2022.12.017

[25] D. K. Breslow, A. J. Holland, Mechanism and regulation of centriole and cilium biogenesis. Annu. Rev. Biochem. 88, 691–724 (2019). 30601682 10.1146/annurev-biochem-013118-111153 PMC6588485

[26] E. A. Nigg, A. J. Holland, Once and only once: Mechanisms of centriole duplication and their deregulation in disease. Nat. Rev. Mol. Cell Biol. 19, 297–312 (2018). 29363672 10.1038/nrm.2017.127 PMC5969912

[27] R. Habedanck, Y.-D. Stierhof, C. J. Wilkinson, E. A. Nigg, The Polo kinase Plk4 functions in centriole duplication. Nat. Cell Biol. 7, 1140–1146 (2005). 16244668 10.1038/ncb1320

[28] T.-S. Kim, J.-E. Park, A. Shukla, S. Choi, R. N. Murugan, J. H. Lee, M. Ahn, K. Rhee, J. K. Bang, B. Y. Kim, J. Loncarek, R. L. Erikson, K. S. Lee, Hierarchical recruitment of Plk4 and regulation of centriole biogenesis by two centrosomal scaffolds, Cep192 and Cep152. Proc. Natl. Acad. Sci. U.S.A. 110, E4849–E4857 (2013). 24277814 10.1073/pnas.1319656110 PMC3864335

[29] K. F. Sonnen, A.-M. Gabryjonczyk, E. Anselm, Y.-D. Stierhof, E. A. Nigg, Human Cep192 and Cep152 cooperate in Plk4 recruitment and centriole duplication. J. Cell Sci. 126, 3223–3233 (2013). 23641073 10.1242/jcs.129502

[30] F. Zhu, S. Lawo, A. Bird, D. Pinchev, A. Ralph, C. Richter, T. Müller-Reichert, R. Kittler, A. A. Hyman, L. Pelletier, The mammalian SPD-2 ortholog Cep192 regulates centrosome biogenesis. Curr. Biol. 18, 136–141 (2008). 18207742 10.1016/j.cub.2007.12.055

[31] M. A. Gomez-Ferreria, U. Rath, D. W. Buster, S. K. Chanda, J. S. Caldwell, D. R. Rines, D. J. Sharp, Human Cep192 is required for mitotic centrosome and spindle assembly. Curr. Biol. 17, 1960–1966 (2007). 17980596 10.1016/j.cub.2007.10.019

[32] L. L. Fava, F. Schuler, V. Sladky, M. D. Haschka, C. Soratroi, L. Eiterer, E. Demetz, G. Weiss, S. Geley, E. A. Nigg, A. Villunger, The PIDDosome activates p53 in response to supernumerary centrosomes. Genes Dev. 31, 34–45 (2017). 28130345 10.1101/gad.289728.116 PMC5287111

[33] M. Burigotto, A. Mattivi, D. Migliorati, G. Magnani, C. Valentini, M. Roccuzzo, M. Offterdinger, M. Pizzato, A. Schmidt, A. Villunger, S. Maffini, L. L. Fava, Centriolar distal appendages activate the centrosome-PIDDosome-p53 signalling axis via ANKRD26. EMBO J. 40, e104844 (2021). 33350486 10.15252/embj.2020104844 PMC7883297

[34] L. T. Evans, T. Anglen, P. Scott, K. Lukasik, J. Loncarek, A. J. Holland, ANKRD26 recruits PIDD1 to centriolar distal appendages to activate the PIDDosome following centrosome amplification. EMBO J. 40, e105106 (2021). 33350495 10.15252/embj.2020105106 PMC7883295

[35] V. Joukov, J. C. Walter, A. De Nicolo, The Cep192-organized aurora A-Plk1 cascade is essential for centrosome cycle and bipolar spindle assembly. Mol. Cell 55, 578–591 (2014). 25042804 10.1016/j.molcel.2014.06.016 PMC4245277

[36] T. Chinen, K. Yamazaki, K. Hashimoto, K. Fujii, K. Watanabe, Y. Takeda, S. Yamamoto, Y. Nozaki, Y. Tsuchiya, D. Takao, D. Kitagawa, Centriole and PCM cooperatively recruit CEP192 to spindle poles to promote bipolar spindle assembly. J. Cell Biol. 220, e202006085 (2021). 33443571 10.1083/jcb.202006085 PMC7812875

[37] J. Holder, J. A. Miles, M. Batchelor, H. Popple, M. Walko, W. Yeung, N. Kannan, A. J. Wilson, R. Bayliss, F. Gergely, CEP192 localises mitotic Aurora-A activity by priming its interaction with TPX2. EMBO J. 43, 5381–5420 (2024). 39327527 10.1038/s44318-024-00240-z PMC11574021

[38] D. C. Zebrowski, S. Vergarajauregui, C.-C. Wu, T. Piatkowski, R. Becker, M. Leone, S. Hirth, F. Ricciardi, N. Falk, A. Giessl, S. Just, T. Braun, G. Weidinger, F. B. Engel, Developmental alterations in centrosome integrity contribute to the post-mitotic state of mammalian cardiomyocytes. eLife 4, e05563 (2015). 26247711 10.7554/eLife.05563 PMC4541494

[39] V. C. Sladky, H. Akbari, D. Tapias-Gomez, L. T. Evans, C. G. Drown, M. A. Strong, G. M. LoMastro, T. Larman, A. J. Holland, Centriole signaling restricts hepatocyte ploidy to maintain liver integrity. Genes Dev. 36, 843–856 (2022). 35981754 10.1101/gad.349727.122 PMC9480857

[40] P. Tátrai, F. Gergely, Centrosome function is critical during terminal erythroid differentiation. EMBO J. 41, e108739 (2022). 35678476 10.15252/embj.2021108739 PMC9289712

[41] A.-K. Weier, M. Homrich, S. Ebbinghaus, P. Juda, E. Miková, R. Hauschild, L. Zhang, T. Quast, E. Mass, A. Schlitzer, W. Kolanus, S. Burgdorf, O. J. Gruß, M. Hons, S. Wieser, E. Kiermaier, Multiple centrosomes enhance migration and immune cell effector functions of mature dendritic cells. J. Cell Biol. 221, e202107134 (2022). 36214847 10.1083/jcb.202107134 PMC9555069

[42] T. Natsume, T. Kiyomitsu, Y. Saga, M. T. Kanemaki, Rapid protein depletion in human cells by auxin-inducible degron tagging with short homology donors. Cell Rep. 15, 210–218 (2016). 27052166 10.1016/j.celrep.2016.03.001

[43] G. M. LoMastro, C. G. Drown, A. L. Maryniak, C. E. Jewett, M. A. Strong, A. J. Holland, PLK4 drives centriole amplification and apical surface area expansion in multiciliated cells. eLife 11, e80643 (2022). 35969030 10.7554/eLife.80643 PMC9507127

[44] L. Zhang, J. D. Ward, Z. Cheng, A. F. Dernburg, The auxin-inducible degradation (AID) system enables versatile conditional protein depletion in C. elegans. Development 142, 4374–4384 (2015). 26552885 10.1242/dev.129635 PMC4689222

[45] M. Trost, A. C. Blattner, C. F. Lehner, Regulated protein depletion by the auxin-inducible degradation system in Drosophila melanogaster. Fly 10, 35–46 (2016). 27010248 10.1080/19336934.2016.1168552 PMC4934730

[46] V. T. Chu, T. Weber, R. Graf, T. Sommermann, K. Petsch, U. Sack, P. Volchkov, K. Rajewsky, R. Kühn, Efficient generation of Rosa26 knock-in mice using CRISPR/Cas9 in C57BL/6 zygotes. BMC Biotechnol. 16, 4 (2016). 26772810 10.1186/s12896-016-0234-4 PMC4715285

[47] X. Tan, L. I. A. Calderon-Villalobos, M. Sharon, C. Zheng, C. V. Robinson, M. Estelle, N. Zheng, Mechanism of auxin perception by the TIR1 ubiquitin ligase. Nature 446, 640–645 (2007). 17410169 10.1038/nature05731

[48] S. Hayashi, P. Lewis, L. Pevny, A. P. McMahon, Efficient gene modulation in mouse epiblast using a Sox2Cre transgenic mouse strain. Mech. Dev. 119, S97–S101 (2002). 14516668 10.1016/s0925-4773(03)00099-6

[49] Y. You, S. L. Brody, Culture and differentiation of mouse tracheal epithelial cells. Methods Mol. Biol. 945, 123–143 (2013). 23097105 10.1007/978-1-62703-125-7_9

[50] J. Xu, Preparation, culture, and immortalization of mouse embryonic fibroblasts. Curr. Protoc. Mol. Biol. 70, 28.1.1–28.1.8 (2005). 10.1002/0471142727.mb2801s70 18265366

