# Supplementary material for: Rapid and sustained degradation of the essential centrosome protein CEP192 in live mice using the AID2 system
Source: Sci Adv. 2025 Feb 28;11(9):eadq2339. doi: 10.1126/sciadv.adq2339 (PMC11870075; doi:10.1126/sciadv.adq2339)
Supplement: Supplementary file 1 — Figs. S1 to S6 [file sciadv.adq2339_sm.pdf]

Science Advances
AAAS

Supplementary Materials for

Rapid and sustained degradation of the essential centrosome protein CEP192 in live mice using the AID2 system

Valentina C. Sladky *et al.*

Corresponding author: Andrew J. Holland, aholland@jhmi.edu

Sci. Adv. **11**, eadq2339 (2025)
DOI: 10.1126/sciadv.adq2339

This PDF file includes:

Figs. S1 to S6

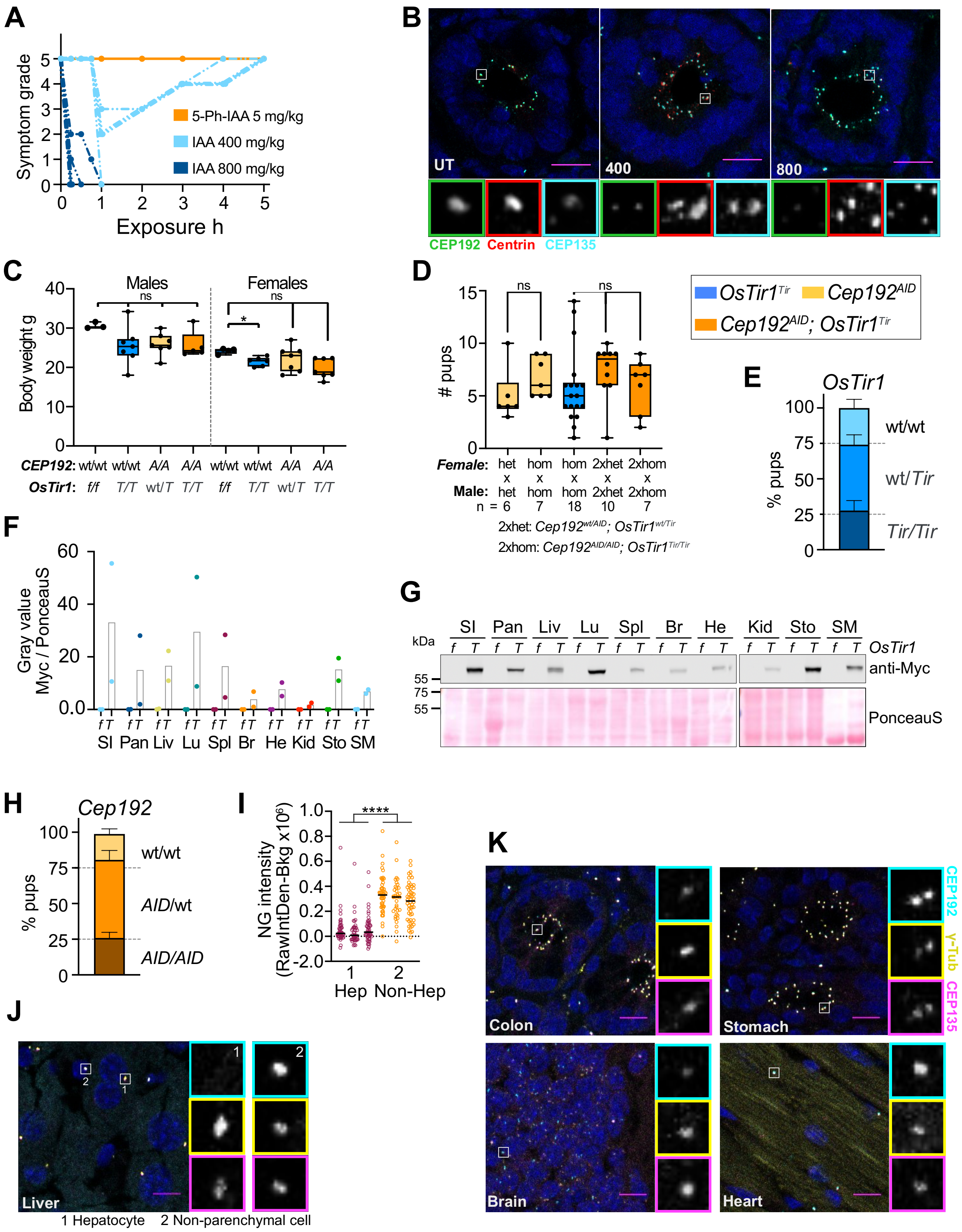

Figure S1. Characterization of the novel *OsTir1*^{Tir} and *Cep192*^{AID} mice.

(**A**) Symptom grades of the individual *OsTir1*$^{Tir/Tir}$ mice injected either with 5mg/kg 5-Ph-IAA (N = 3), 400mg/kg IAA (N = 5), or 800mg/kg IAA (N = 5) as in Fig. 1B. Symptoms including spasms

and paralysis were graded by severity over time; 5 = symptom-free, 0 = near complete paralysis, humane endpoint.

(**B**) Confocal immunofluorescence images showing CEP192^{AID} (NeonGreen; green), and immunostained Centrin (red) and CEP135 (cyan) in the SI of OSTIR1 expressing mice that were left untreated or injected with 400mg/kg of IAA for 5h or 800mg/kg of IAA for 1h. Scale bars 10 µm.

(**C**) Graph showing the body weight of 9-30 weeks old male and female mice of the indicated genotype combinations. *Cep192*: A/A – AID/AID, *OsTir1*: f/f – floxed/floxed, T/T – Tir/Tir. N = 3-8 mice per genotype.

(**D**) Litter sizes of breedings with the indicated genotype combinations of homozygous (hom) or heterozygous (het) *Cep192*^{AID} and *OsTir1*^{Tir}alleles. n = 6-18 litters as noted in the figure. 2xhet: *Cep192*$^{wt/AID}$; *OsTir1*$^{wt/Tir}$. 2xhom: *Cep192*$^{AID/AID}$; *OsTir1*$^{Tir/Tir}$.

(**E**) Genotype distribution of the offspring of *OsTir1*^{Tir} heterozygous breedings. Dashed lines mark the expected Mendelian distribution. N = 9 litters.

(**F**) Quantification of the immunoblots shown in Fig. 1D and in (G) showing the expression of OSTIR1-F74G-Myc relative to total protein loaded (PonceauS) across tissues in *OsTir1*$^{f/f}$ *(f)* and an *OsTir1*$^{Tir/Tir}$ *(T)* mice. N = 2 mice per genotype.

(**G**) Immunoblot probed with an antibody detecting OSTIR1-F74G-Myc in the indicated organs of an *OsTir1*$^{f/f}$ *(f)* and an *OsTir1*$^{Tir/Tir}$ *(T)* mouse. The experiment is a biological replicate of the immunoblot shown in Fig. 1D. SI – small intestine, Pan – Pancreas, Liv – Liver, Lu – Lung, Spl – Spleen, Br – Brain, He – Heart, Kid – Kidney, Sto – Stomach, SM – Skeletal Muscle. PonceauS-staining is shown as a reference for the amount of protein loaded.

(**H**) Graph showing the genotype distribution of the offspring of heterozygous *Cep192*^{AID} breedings. Dashed lines mark the expected Mendelian distribution. N = 11 litters.

(**I**) Quantification of the CEP192^{AID} NeonGreen (NG) signal in hepatocytes (1) and non-parenchymal cells (2, non-Hep) in the liver as in (I-J). Scale bar 10 µm.

(**J**) Representative confocal immunofluorescence image of the liver showing the CEP192^{AID} signal (NeonGreen, cyan), and immunostained γ-tubulin (yellow) and CEP135 (magenta) in hepatocytes (1) and non-parenchymal cells (2, non-Hep). Scale bar 10 µm.

(**K**) Representative confocal immunofluorescence image of the indicated organs showing the CEP192^{AID} signal (NeonGreeen, cyan), and immunostained γ-tubulin (yellow) and CEP135 (magenta). Scale bar 10 µm.

Data is displayed as mean ± SEM. Statistical significance was determined by one-way ANOVA with Sidak's multiple comparisons test. In (A), male and female groups were analyzed separately. ns $p \geq 0.05$, * $p<0.05$, **** $p<0.0001$.

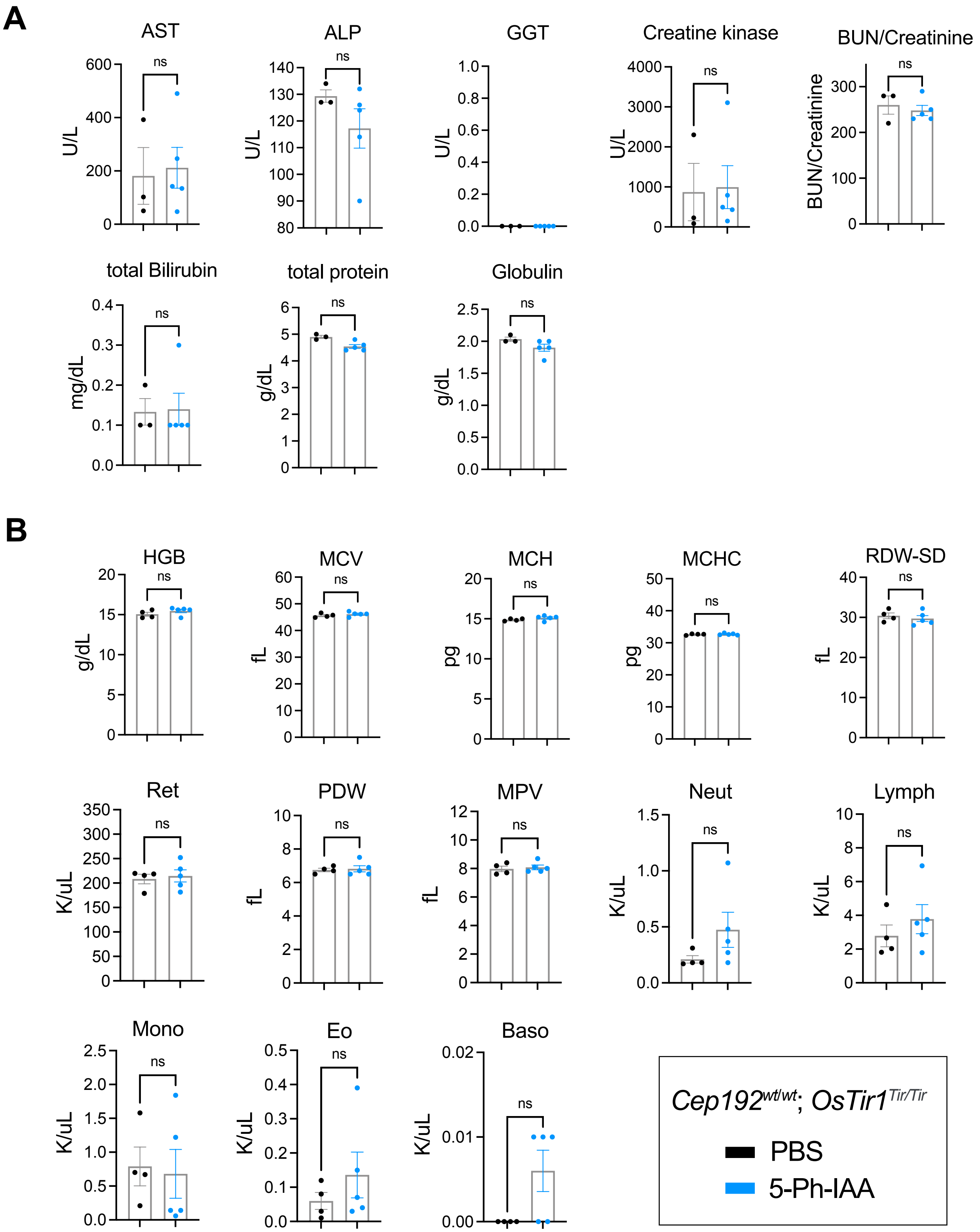

Figure S2. Repeated dosing with 5-Ph-IAA has no impact on blood and serum parameters. (**A-B**) Analysis of serum and blood parameters of *OsTir1*$^{Tir/Tir}$ mice treated with 5-Ph-IAA or PBS every 24h for 14 days.

(**A**) Graphs showing the serum parameters. AST – Aspartate aminotransferase, ALP – Alkaline phosphatase, GGT – Gamma-glutamyltransferase, BUN – Blood urea nitrogen.

(**B**) Graphs showing blood cell characterization. HGB – Hemoglobin, MCV – Mean corpuscular volume, MCH – Mean corpuscular hemoglobin, MCHC – Mean corpuscular hemoglobin concentration, RDW-SD – Red cell distribution width, Ret – Reticulocytes, PDW – Platelet distribution width, MPV – Mean platelet volume, Neut – Neutrophils, Lymph – Lymphocytes, Mono – Monocytes, Eo – Eosinophils, Baso – Basophils.

Data is displayed as mean ± SEM. Statistical significance was assessed by two-tailed, unpaired Student's t-test. ns $p \geq 0.05$.

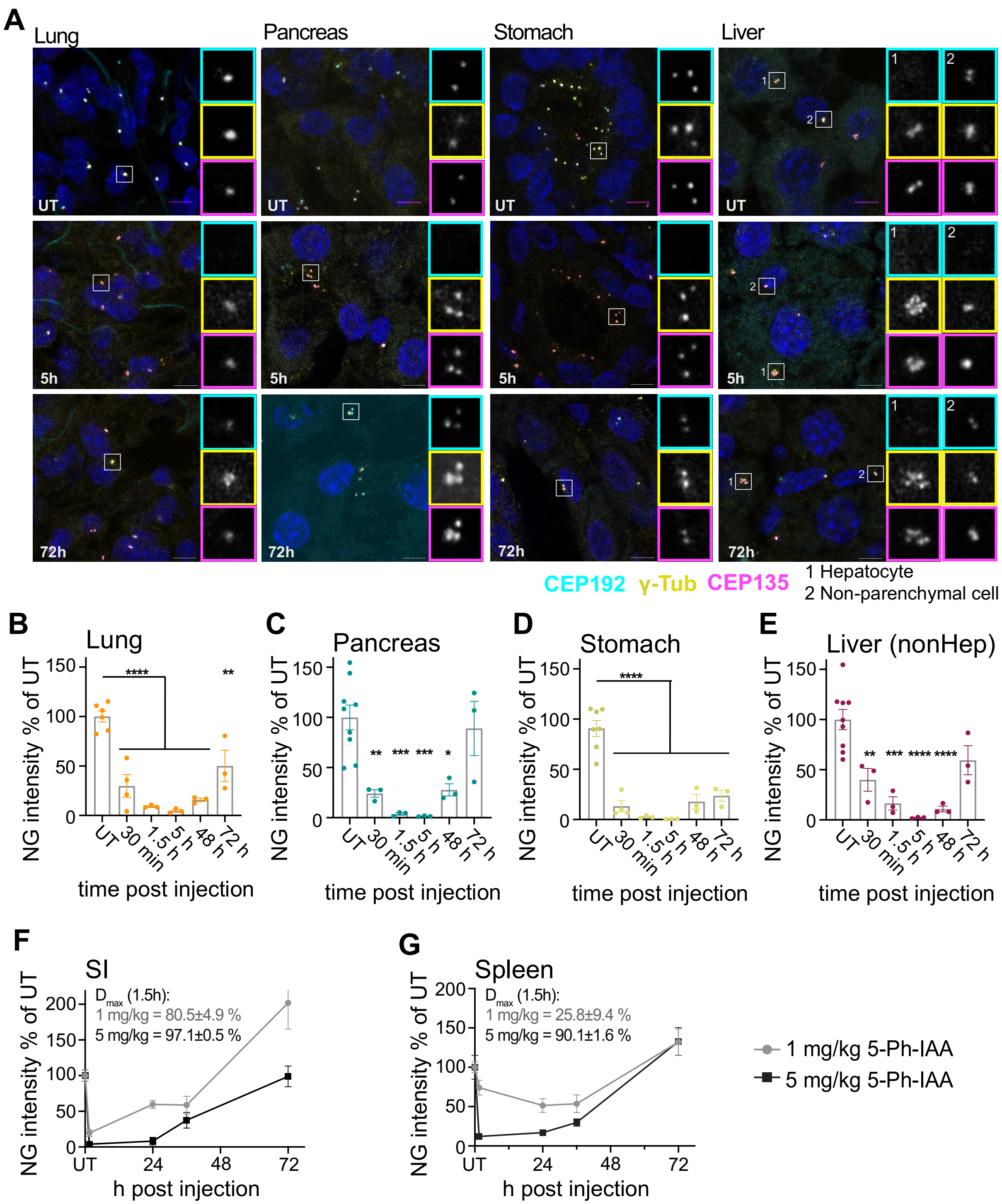

Figure S3. 5-Ph-IAA dose can alter the degradation dynamics.

(**A**) Representative immunofluorescence images of the indicated organs from *Cep192*$^{AID/AID}$ mice expressing OSTIR1 that were untreated (UT) or injected with 5-Ph-IAA for 5 and 72 hours. CEP192^{AID} signal (cyan), and immunostained γ-tubulin (yellow) and CEP135 (magenta); scale bars 5 µm.

(**B-E**) Quantification of the $CEP192^{AID}$ NeonGreen (NG) signal relative to the untreated control (UT) of (**B**) lung, **(C)** pancreas, **(D)** stomach, and **(E)** non-parenchymal liver cells (NonHep) isolated from $\textit{Cep192}^{AID/AID}$ mice expressing OSTIR1 at the indicated time points after 5-Ph-IAA injection. N = 3-9 mice per timepoint, n = 40-100 cells per mouse.

(**F-G**) Graphs show the $CEP192^{AID}$ NeonGreen (NG) signal relative to the untreated control (UT) of the **(F)** SI and **(G)** spleen of $\textit{Cep192}^{AID/AID}$; $\textit{OsTir1}^{Tir/Tir}$ mice analyzed at 1.5h, 24h, 36h, and 72h after injection of 1 mg/kg or 5 mg/kg 5-Ph-IAA. N = 3-5 mice per timepoint, n = 50-100 cells per mouse.

Data is shown as mean ± SEM. Statistical significance was determined using a one-way ANOVA with Sidak's multiple comparisons test comparing each timepoint to the untreated control, * $p<0.05$, ** $p<0.01$, *** $p<0.001$, **** $p<0.0001$. Only significant results are indicated.

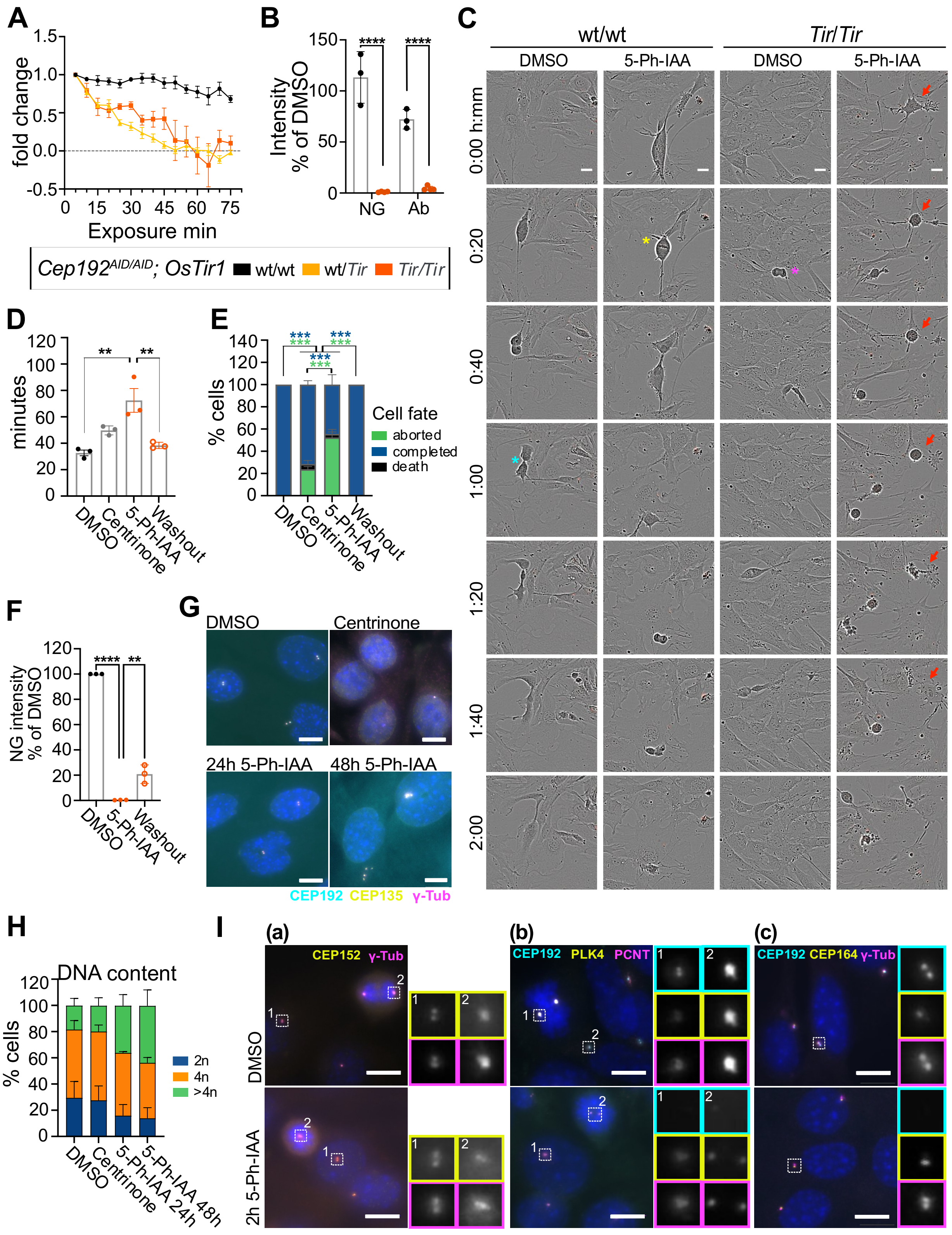

Figure S4. Complete degradation of CEP192^{AID} in MEFs impairs mitosis.

(**A**) Graph showing the CEP192^{AID} NeonGreen signal intensity measured relative to timepoint t = 0 min in primary MEF lines of the indicated genotypes. 5-Ph-IAA was added at timepoint t = 0

min. *Cep192*$^{wt/AID}$; *OsTir1*$^{wt/wt}$ N = 1 MEF lines, n = 4 cells; *Cep192*$^{wt/AID}$; *OsTir1*$^{wt/Tir}$ N = 2, n = 3-8; *Cep192*$^{wt/AID}$; *OsTir1*$^{Tir/Tir}$ N = 3, n = 3-11.

(**B**) Primary *Cep192*$^{AID/AID}$; *OsTir1*$^{Tir/Tir}$ and *Cep192*$^{AID/AID}$; *OsTir1*$^{wt/wt}$ MEF lines treated with 5-Ph-IAA for 5h were immunostained with an antibody raised against mouse CEP192 and an antibody recognizing γ-tubulin. Signal intensities of CEP192^{AID} (NeonGreen), and immunostained CEP192 and γ-tubulin were quantified relative to the DMSO condition. N = 3-4 MEF lines per genotype, n = 30-50 cells per MEF line.

(**C**) Representative live imaging brightfield stills of mitotic primary *Cep192*$^{AID/AID}$ MEFs with or without OSTIR1 treated with 5-Ph-IAA or DMSO. MEFs without OSTIR1 or 5-Ph-IAA undergo complete mitosis with visible metaphase plates (yellow *), anaphase (magenta *), and cytokinesis (cyan *). MEFs expressing OSTIR1 and treated with 5-Ph-IAA round-up and re-adhere to the plate without undergoing cell division (red arrow).

(**D-F**) SV40-immortalized *Cep192*$^{AID/AID}$; *OsTir1*$^{Tir/Tir}$ MEFs were live imaged to determine the duration and fate of mitosis following DMSO, Centrinone, and 5-Ph-IAA treatments. MEFs were pre-treated with centrinone for 24h to deplete centrioles before imaging for 48h. DMSO and 5-Ph-IAA were added at t = 0h for the entire imaging time of 48h. For the washout condition, 5-Ph-IAA was added at t = 0h and replaced with fresh media at t = 24h to image recovering cells for another 24h. (**D**) Quantification of the time spent in mitosis from rounding up until completion of cell division or cell death. (**E**) Graph showing the cell fate after mitosis: cells completed mitosis by successful division (blue), died (black), or re-adhered without division (green). (**F**) CEP192^{AID} signal intensity was quantified in immunofluorescence microscopy images of the indicated conditions. n = 50-100 mitotic cells per N = 3 MEF lines.

(**G**) Representative immunofluorescence microscopy images of SV40-immortalized *Cep192*$^{AID/AID}$; *OsTir1*$^{Tir/Tir}$ MEFs treated as in (D-E). Cells were fixed, stained and imaged after completion of the live imaging experiment. CEP192^{AID} (cyan), γ-tubulin (magenta) and CEP135 (yellow). Scale bars 10 µm.

(**H**) Stacked bar graph showing the DNA content distribution measured by flow cytometry of SV40-immortalized *Cep192*$^{AID/AID}$; *OsTir1*$^{Tir/Tir}$ MEFs treated with DMSO, Centrinone (48h), and 5-Ph-IAA (24h, 48h). N = 3 MEF lines.

(**I**) Representative immunofluorescence images of SV40-immortalized *Cep192*$^{AID/AID}$; *OsTir1*$^{Tir/Tir}$ MEFs treated with DMSO or 5-Ph-IAA for 2h (related to Fig. 4H-L). The cells were immunostained with antibodies detecting the indicated centrosomal proteins. CEP192^{AID} is shown in cyan. (**a**) CEP152 (yellow), γ-tubulin (magenta). (**b**) CEP192^{AID} (cyan), PLK4 (yellow), PCNT (magenta). (**c**) CEP192^{AID} (cyan), CEP164 (yellow), γ-tubulin (magenta).

All data is shown as mean ± SEM. Statistical significance was measured by one-way ANOVA (B-D) or two-way ANOVA (E, G) with Sidak's multiple comparisons test (B). ns $p \geq 0.05$, ** $p<0.01$, *** $p<0.001$, **** $p<0.0001$. Only significant results are indicated.

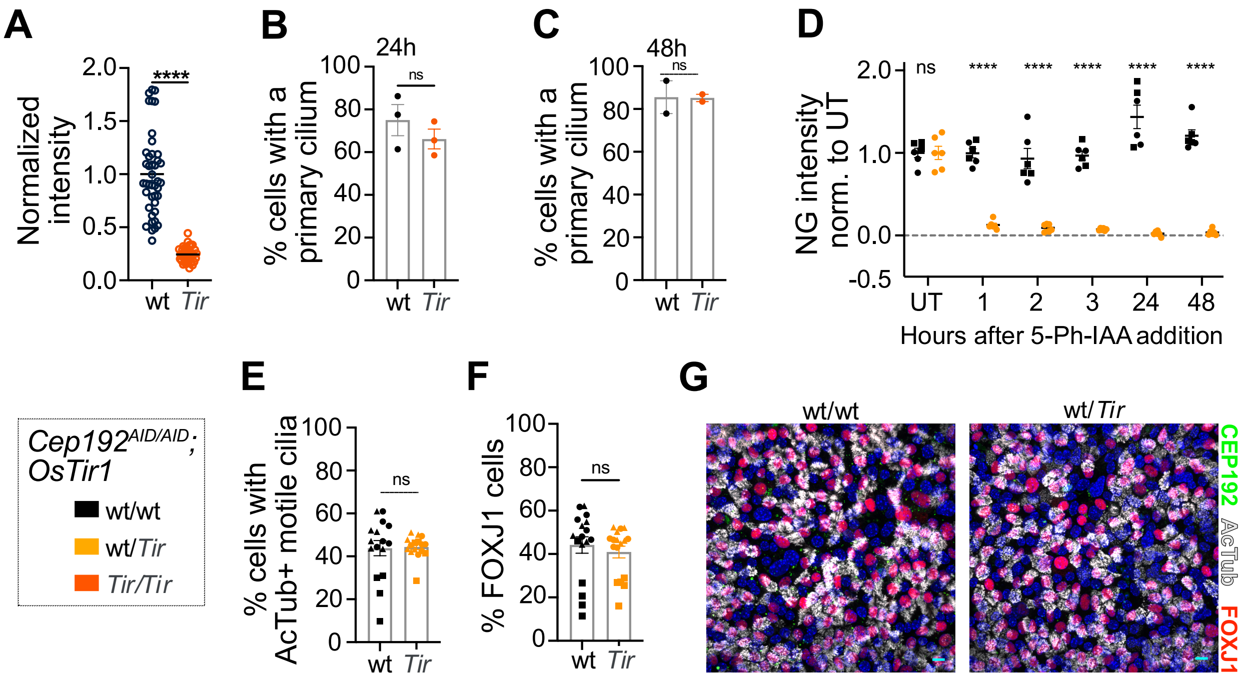

Figure S5. CEP192 is not required for primary or motile cilia maintenance

(**A**) Quantification of the NeonGreen signal of serum-starved *Cep192$^{AID/AID}$* MEFs with or without OSTIR1 normalized to the mean of *Cep192$^{AID/AID}$*; *OsTir1$^{wt/wt}$* MEFs. n = 39-42 cells per genotype.

(**B-C**) Quantification of *Cep192$^{AID/AID}$* MEF cells wt/wt or wt/Tir for *OsTir1* with a primary cilium. Cells were serum starved for 24 h before 5-Ph-IAA treatment for (**B**) 24h or (**C**) 48h. N = 2 MEF lines per genotype from 1 or 2 separate passages.

(**D**) Time course of 5-Ph-IAA treatment of mTEC cultures showing CEP192^{AID} (NeonGreen) intensity at the centrioles of non-multiciliated cells without or with OSTIR1 (wt/*Tir*) relative to untreated control. N = 2 mice per genotype indicated by symbol shape, n = 6 fields of view.

(**E-G**) mTECs were cultured at an air-liquid-interphase (ALI) for 7 days to allow motile cilia formation. 5-Ph-IAA was added to the basal media for ALI days 7-9. (**E**) Quantification of the percentage of cells with motile cilia marked by acetylated tubulin (AcTub) and (**F**) the fraction of cells positive for the differentiation marker FOXJ1. N = 3 mice per genotype indicated by symbol shape, n = 6 fields of view. (**G**) Representative confocal images of mTEC cultures of the indicated genotypes expressing CEP192^{AID} (green) and immunostained for AcTub (gray) and FOXJ1 (red). Scale bars 10 µm.

All data is shown as mean ± SEM. Statistical significance was measured by two -way ANOVA with Sidak's multiple comparisons test (D), and two-tailed, unpaired Student's t-test (A-C, E-F). ns p≥0.05, **** p<0.0001.

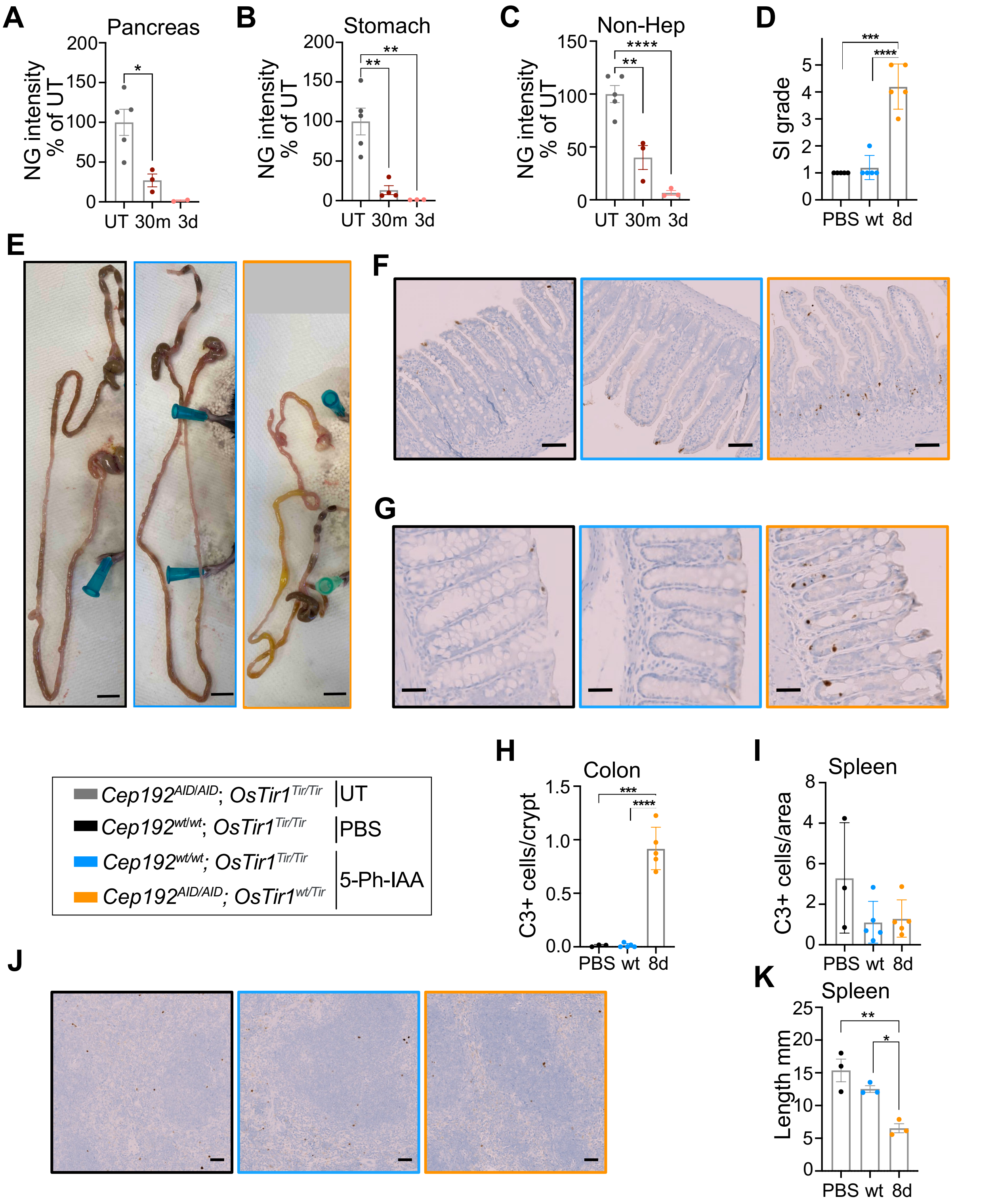

Figure S6. Sustained degradation of CEP192^{AID} causes gastrointestinal symptoms and cell death.

(**A-C**) *Cep192*$^{AID/AID}$ mice expressing OSTIR1 were injected with 5 mg/kg 5-Ph-IAA. Analysis performed 30 min after 5-Ph-IAA administration or following repeat 5-Ph-IAA administration every 12 h for 3 days. CEP192^{AID} signal intensity (NeonGreen, NG) was quantified in fluorescence microscopy images relative to the untreated (UT) control in (**A**) the pancreas (N = 2-5), (**B**) the stomach (N = 3-5) and (**C**) in non-parenchymal liver cells (N = 3-5) at the indicated timepoints.

(**D**) Graph showing the severity of the gastrointestinal symptoms in mice after 8 days of repeated 5-Ph-IAA or PBS injections. Symptoms were graded 1-5 based on stool consistency and color in the small intestine. 1 = normal stool; 5 = watery liquid, yellow stool. N = 3-5 mice per genotype and treatment.

(**E**) Example images for (D) showing SI grade 1 and 5 symptoms. Images from *Cep192*$^{wt/wt}$; *OsTir1*$^{Tir/Tir}$ mice treated with PBS (black box) or 5-Ph-IAA (blue box) and *Cep192*$^{AID/AID}$; *OsTir1*$^{wt/Tir}$ exposed to 5-Ph-IAA (orange box). Scale bar 1cm.

(**F-G**) Cell death in the crypts of the SI and the colon was measured using immunohistochemistry for cleaved caspase-3 (C3). Representative images of the (**F**) SI and the (**G**) colon of *Cep192*$^{wt/wt}$; *OsTir1*$^{Tir/Tir}$ mice treated with PBS (black box) or 5-Ph-IAA (blue box), and *Cep192*$^{AID/AID}$; *OsTir1*$^{wt/Tir}$ mice injected with 5-Ph-IAA (orange box) for 8 days. Scale bars 100 µm. N = 3-5 mice per genotype and condition.

(**H**) Graph showing the number of cleaved caspase-3 positive cells per crypt in the colon.

(**I**) Quantification of cleaved caspase-3 positive cells in the spleen per area. Cell death was measured by immunohistochemistry for cleaved caspase-3 (C3). N = 3-5 mice per genotype and condition.

(**J**) Representative images of cleaved caspase-3 immunohistochemistry staining on spleens of *Cep192*$^{wt/wt}$; *OsTir1*$^{Tir/Tir}$ mice injected with PBS (black box) or 5-Ph-IAA (blue box), and *Cep192*$^{AID/AID}$; *OsTir1*$^{wt/Tir}$ exposed to 5-Ph-IAA (orange box) for 8 days. Scale bars 100 µm.

All data is shown as mean ± SEM. Statistical significance was measured using one-way ANOVA with Sidak's multiple comparisons test. Of note, no statistical analysis was performed for (A) 3d, since this timepoint includes only N = 2 mice. Only significant results are indicated. ns $p \geq 0.05$, * $p<0.05$, ** $p<0.01$, *** $p<0.001$, **** $p<0.0001$.
